# Discrepancy and Disliking Do Not Induce Negative Opinion Shifts

**DOI:** 10.1371/journal.pone.0157948

**Published:** 2016-06-22

**Authors:** Károly Takács, Andreas Flache, Michael Mäs

**Affiliations:** 1 MTA TK “Lendület” Research Center for Educational and Network Studies (RECENS), Hungarian Academy of Sciences, Budapest, Hungary; 2 Department of Sociology/ICS, University of Groningen, Groningen, The Netherlands; Université Toulouse 1 Capitole, FRANCE

## Abstract

Both classical social psychological theories and recent formal models of opinion differentiation and bi-polarization assign a prominent role to negative social influence. Negative influence is defined as shifts away from the opinion of others and hypothesized to be induced by discrepancy with or disliking of the source of influence. There is strong empirical support for the presence of positive social influence (a shift towards the opinion of others), but evidence that large opinion differences or disliking could trigger negative shifts is mixed. We examine positive and negative influence with controlled exposure to opinions of other individuals in one experiment and with opinion exchange in another study. Results confirm that similarities induce attraction, but results do not support that discrepancy or disliking entails negative influence. Instead, our findings suggest a robust positive linear relationship between opinion distance and opinion shifts.

## Introduction

Social influence is a powerful force that fosters opinion convergence in groups [1–4]. People assimilate their views to real or perceived opinions of others [5–9]. Repeated social influence in groups, organizations, and societies should, therefore, result in a gradual decrease in opinion variance. In fact, mathematical models demonstrated that when people consistently move their opinions closer to the opinions of those they interact with, perfect opinion *consensus is inevitable*, unless some subset of group members is entirely cut off from interaction [10–16]. This has left social scientists studying social influence with a theoretical puzzle of why there is persistent opinion diversity in the presence of permanent social influence [9, 12, 17].

Neither small groups, organizations, neighborhoods, or society at large exhibit an inevitable tendency towards perfect consensus, as examples from group discussion experiments as well as studies of political, social and cultural views demonstrate [18–23]. Studies of college dormitories [24], international work teams [25], and representative opinion surveys on controversial issues in the public debate [26–28] even demonstrated that influence dynamics sometimes result in gradually *increasing* dissimilarity and bi-polarization [29].

Researchers have proposed the *negative influence hypothesis* as a possible explanation. Under certain conditions individuals adjust their opinions in such a way as to become more dissimilar to others they disagree with [9]. Here, we report results from two experiments that tested whether large opinion distance could indeed trigger opinion shifts away from the source. Formal models of opinion dynamics have demonstrated that with this type of negative influence assumed, one can reconcile social influence with the observation of persisting and sometimes even increasing opinion variation in a population [9, 19, 30–37]. Balance theory [38], cognitive dissonance theory [39], and social judgment theory [40] have been used to justify that positive and negative social influence must be differentiated, assuming that people strive for agreement with a person who is similar and for disagreement with persons who are distant.

To date, however, empirical research has not provided unequivocal empirical support for this negative influence hypothesis, despite its prominent role in the social psychology literature [41]. Some experimental support for the negative influence assumption comes from studies based on self-categorization theory [42] that tested whether exposure to perceived out-group opinions may increase opinion differences between in- and out-group opinions. Furthermore, studies that supported the negative influence hypothesis have been criticized for various shortcomings [29, 41]. First, some existing designs did not separate positive influence from the in-group and negative influence from the out-group in the explanation of opinion shifts [42–44]. In these studies, participants were exposed to opposing opinions of out-group members and opinions of in-group members that were similar to the opinion of the target participant but more extreme. Participants did shift opinions away from the opinion of the out-group [45], but it is not clear whether these were caused by negative influence from the out-group or positive influence from more extreme in-group members. Second, studies where initial dissimilarity was measured as perceived distance do not allow concluding whether observed opinion shifts resulted from opinion dissimilarity or from disliking arising from perceived differences on other dimensions [45–46]. Third, field experiments that extended over a longer time span did not control for general opinion trends that occurred in parallel with the shifts induced by the experimental manipulation [47–48]. Finally, existing designs cannot readily disentangle whether shifts away from a source of influence may be induced by perceived dissimilarity to the source, or by disliking of a negatively perceived source, as suggested by [49].

The lack of conclusive evidence for assessing the negative influence hypothesis and the shortcomings of earlier studies provided the motivation for our two laboratory experiments. We designed experiments that differed in several ways from existing designs. We avoided group terminology, never revealed salient characteristics of the source of influence, and investigated opinion shifts in a single opinion dimension, as these factors might complicate the relationship between discrepancy and opinion shifts directly or via attraction towards the source [42–46, 48–51].

Most importantly, however, we studied influence in a highly controlled environment that allowed us to differentiate between the confounding effects of attraction and opinion similarity. In Study 1, we *measured* attraction towards the source purely based on opinion differences. We conceptualized attraction as likeability and measured it on a positive scale from 0 to 100, where zero was labeled as “very much disliking” and one-hundred as “very much liking”. By the exclusion of other factors that might influence attraction, we tested how initial opinion differences cause opinion shifts. In Study 2, we *manipulated* the degree to which participants liked or disliked the source of influence, to test whether attraction confounds with effects of initial opinion differences on opinion shifts. In both studies, we measured opinions on a 0…100 scale such that both the direction and magnitude of opinion change could be assessed. In addition, both studies were designed to avoid potential shortcomings of previous research and had the following core design features. First, participants were informed about the opinion of only one other participant of the experiment at a time, to assure that opinion shifts were not caused by multiple and potentially conflicting sources of influence. Second, to be able to statistically control for general trends in opinions, we measured participants’ opinions before and after being exposed to the opinions of others without any intermediate exposure to other sources of influence. Third, the experiments were designed to avoid that participants would perceive the situation in inter-group terms. Fourth, participants could not themselves select the source of influence and were placed in a highly controlled setting. Participants were aware that their financial compensation was not based on their responses. In this way, alternative explanations of influence and attraction dynamics that are based on financial motives, on need for consensus, on endogenous interaction dynamics, on argumentation, on persuasive power, on source credibility [50–51], or on perceived threat could be excluded.

## Hypotheses

### Positive shifts

**Fig 1:**
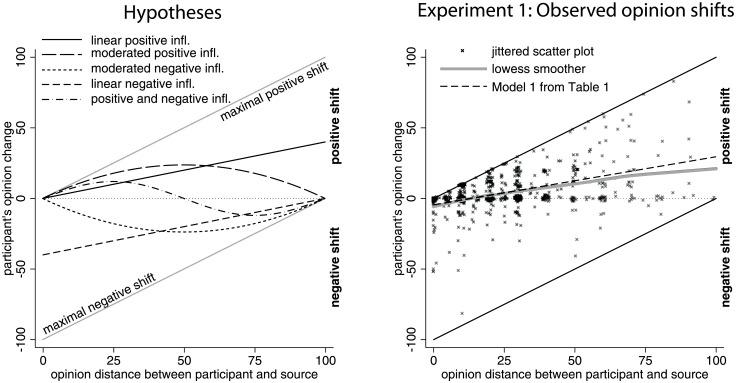
Visualization of the hypotheses and observed opinion shifts during Study 1. The horizontal axis of Fig 1 shows initial opinion distance, which—following specifications of positive and negative influence in recent formal modeling work—is the core independent variable of our analyses. The vertical axis charts the main dependent variable, the direction and magnitude of opinion change towards the source after the first stimulus. Positive values indicate that the participant’s opinion shifted towards the source. Negative values represent an opinion shift away from the source. The magnitude measures in both cases how much the initial opinion dissimilarity was reduced or increased by the opinion shift in absolute terms. The two grey solid lines define the maximal positive and negative opinion shift that is possible given the initial opinion distance.

The left panel of Fig 1 illustrates our hypotheses about the effect of opinion discrepancy, operationalized as initial distance to the opinion of the source on the direction and magnitude of the resulting opinion shift on the given issue. The *linear positive influence hypothesis* predicts that individuals always decrease differences between their own opinion and the opinion of the source of influence. A positive opinion shift that is *linear* in the original opinion-distance corresponds to the earliest and simplest formal models of social influence [10, 13–14] and follows from the assumption that individual opinions are updated as a weighted average of the opinions of relevant others. The larger the previous distance to the source, the larger is the absolute shift towards the opinion of the source. The linear positive influence hypothesis is illustrated by the linear solid line in the left panel of Fig 1.

The linear positive influence hypothesis has been criticized for assuming that opinion shifts are the strongest when individuals completely disagree. Alternatively, it has been proposed that influence is weak when the discrepancy between the opinions is large, either because of lack of interest or insuperable distance in communicating the position [51]. The notion that positive influence is limited when initial differences are big on the given issue is represented by the *moderated positive influence hypothesis*. This hypothesis predicts an inverted U-shaped effect of initial opinion distance on the magnitude of positive opinion shift towards the source (see Fig 1). Predicted opinion shifts are small when the individual strongly disagrees with the source. Furthermore, predicted opinion shifts are also small in magnitude when opinion differences are small, because small differences logically exclude large shifts towards the source. Expected shifts are biggest when opinions differ enough to allow for a large shift towards the source’s position, but the discrepancy is not too large to substantially weaken influence. Confirmation for this hypothesis could be obtained if the relationship between initial opinion distance and opinion shift is better described by the combination of a linear and quadratic parameter rather than by a simple linear effect of distance, such that overall an inverted U-shaped relationship is predicted (see Fig 1).

In addition, large opinion differences might decrease *attraction* towards the source, which in turn might limit positive influence. In parallel, small opinion differences might trigger tendencies of *homophily*: liking those who are similar to you and increasing the likelihood of interacting with them. Four decades of research in the attraction paradigm showed that the larger the similarity is, the larger the liking of the source [52–53]. It has also been found that attraction triggers convergence towards the opinion of the source directly or indirectly via the increased relevance of the source [40, 48, 54–57]. Similarly, several formal models took into account the positive reinforcing dynamics of similarity and attraction and assumed that positive influence is limited when individuals dislike each other or are dissimilar [19, 58–62]. In Study 2, we therefore manipulated attraction independently, allowing to separating its main effect from the effect of opinion distance.

In order to further examine whether attraction towards the source mediates the effect of initial opinion distance on opinion shift, we also tested the underlying *attraction hypothesis* that opinion similarity induces attraction towards the source of influence. Byrne’s work has in particular specified the attraction hypothesis as a *linear* relationship between similarity and attraction [52–53].

### Negative shifts

Negative shift occurs when individuals change their opinions in a way as to *increase* opinion differences with the source. Opinions might be rejected and contrasted if they are too discrepant and fall into the latitude of rejection [63]. In accordance with the linear positive influence hypothesis, the simplest theoretical possibility describes shifts away from the source as a linear effect of opinion distance (*linear negative influence hypothesis*, see Fig 1).

Shifts away from the source of influence can be argued from the position of cognitive dissonance theory [64–65]. Individuals, it is argued, reduce dissonance created by the discrepancy of opinions by moving their opinions away from a disliked source. Beyond a certain point, additional discrepancy between a position recommended by the source and the initial position of the target may decrease persuasion [12, 57, 65]. Other studies have found that a dissimilar communicator may even evoke a “boomerang effect” [37, 66–67] where information from dissimilar others causes inverted attitude change [40, 68]. Theories that link discrepancy to negative opinion change imply that, all other things being equal, beyond a certain critical level of opinion discrepancy, more dissimilarity induces a larger shift away from the source [30, 35].

The magnitude of negative opinion shifts is logically constrained in a bounded opinion interval. The more dissimilar opinions are, the less room is for even more dissimilarity. This implies a nonlinear shape of negative influence, which is the mirror image of the moderated positive influence hypothesis. The *moderated negative influence hypothesis* predicts that when the initial opinion distance increases, this induces an increasingly larger opinion shift away from the source on the given issue if the discrepancy is low. But beyond a tipping point generated by the interplay of increasing need of distancing and decreasing room for shifts away from the source, more initial dissimilarity reduces the magnitude of negative opinion shift.

None of the theories or empirical studies informing negative social influence claimed that influence is only negative. The *positive and negative influence hypothesis*, therefore, combines the two forms of influence. According to the *positive and negative influence hypothesis*, more similarity induces a larger relative positive opinion shift when initial distance is relatively low such that influence is positive but moderated by dissimilarity in this region of the distance space. Beyond a critical level of distance, less similarity induces a negative opinion shift. This negative shift increases first with larger discrepancy. But due to the constraints of the opinion scales, room for negative shifts is limited when the discrepancy becomes too large. Taken together, this generates the prediction of the wave-shaped pattern on the given issue shown in Fig 1. This pattern can be tested with inclusion of a linear, a quadratic, and a cubic distance term in the explanatory model.

Discrepancy could also have an effect on opinion dynamics via the mechanism of derogating the source [69]. Disliking or derogating the source evolves more likely when individuals have a larger disagreement [70–74]. In line with this argument, in Study 2, we examine whether attraction (disliking) moderates the effect of opinion distance on opinion shifts. As part of checking for this moderation, we test the *repulsion hypothesis*, according to which opinion dissimilarity reduces attraction towards the source of influence and leads to feelings of disliking [71–73, 75–77].

## General Method

For our experiments, we selected issues that covered a wide range of topics as we were interested in *general tendencies* in opinion dynamics and not in context-specific explanations. Participants’ opinions were measured on a 101 point “percentage” scale ranging from 0% to 100%. At the very beginning of their session, participants indicated their initial opinions on 31 issues in Study 1 and 20 issues in Study 2. All issues as well as descriptive statistics are listed in S1 Table. We selected these issues from a list of 83 issues in pilot studies. In particular, we chose those issues that pilot participants identified as clear and salient. Furthermore, we focused on issues where opinion *variance was high* and where opinions did not strongly cluster on round numbers. The selected issues did not have an objectively true opinion value (unlike in [5, 51]) and were topics where social-desirability effects and “cultural truism” effects [69] are unlikely. Our *attraction* measure was adopted from Byrne’s classical work [52: 427]. That is, we asked participants: “We would like to know your feelings about how much would you probably like this person”. The answering scale ranged from 0 to 100, where zero was labeled as “very much disliking” and one-hundred as “very much liking”.

The experimental design has been approved by the Ethics Committee of the Department of Sociology at the University of Groningen. Informed consent in a written form has been obtained from all participants. The data were analyzed anonymously.

## Study 1

### Procedure

The experiment took place at the University of Groningen and lasted 45 minutes per session. Participants were randomly seated in cubicles and responded to a web-based questionnaire. They first rated their opinions on 31 issues and indicated how important they considered each issue. Next, for every participant an issue was selected. Participants were exposed to the opinion of the source on the selected issue (*first stimulus*). This opinion of the source was drawn from a pilot or an earlier session, which allowed using a web-based questionnaire without real online interactions. Further details about the procedure can be found in S1 Text. As all pre-selected issues had a relatively high variance of opinions (S1 Table), our matching method ensured that we obtained a wide range of opinion similarity.

After the *first stimulus*, we measured participants’ attraction towards the source (the other person) and assessed their opinions a second time on the same screen. Given the lack of any other information, participants could only base the attraction rating on the initial opinion of the source (the first stimulus). Participants’ attraction towards the source and the difference between the second opinion measure and the opinion at the very beginning of the experiment constituted the two dependent variables of our analyses. Details of the measurement of the dependent variables can be found in S2 Text.

Subsequently, participants were informed about the updated opinion of the source on the issue (*second stimulus*, S1 Text), followed by another measurement of attraction and opinion change. Here, we focus on the effects of the first stimulus. The effects of the second stimulus were very much in line with those of the first, although the effects of the second stimulus turned out to be weaker.

For each participant, this procedure was repeated 7 to 9 times. Each time, a new source and a new issue was chosen. The order of issues followed an automated selection procedure that avoided repetition and spread issues evenly across different opinion positions. This manipulation created variance in opinion distance between the target and source, which is the core independent variable of our study. At the end of the experiment, participants completed a questionnaire asking background data and motivations during the experiment.

### Participants

Participants were 108 first and second year students of sociology at the University of Groningen (in the Netherlands) who participated as study requirement. After excluding all students from the pilot study, *N* = 89 participants were included in the analyses. As every participant was exposed to multiple sources, the total number of observed opinion exchanges was 617.

### Results

#### Effects on opinion shifts

S1 Fig displays opinion shifts after the first stimulus on the absolute opinion scale, while the right panel in Fig 1 illustrates how opinions shifted, depending on the initial opinion distance between the target and the source. The scatter plot shows that there were opinion shifts that resulted in increased opinion differences. These negative shifts, however, were observed mainly when initial opinion differences were very small and positive shifts were hardly possible. The lowess-smoother curve fitted to the scatter plot shows the average association between the two variables across the 617 observations. It shows that the relationship between opinion distance and opinion shift was *positive* and rather *linear*, in line with the *linear positive influence hypothesis*. On average, the larger the initial opinion discrepancy, the larger the opinion shift *towards* the opinion of the source.

As more careful tests, we estimated random intercept (multilevel) regression models [78–79] with opinion change after the first stimulus as the dependent variable (see Table 1). We estimated random intercept (multilevel) regressions, because opinions were nested in subjects. Each participant was exposed to multiple sources in the experiment and is, thus, represented multiple times in our dataset, which violates the assumption of standard statistical tests that observations are independent. Our core independent variable was the initial opinion distance between the participant and the source. We also included a quadratic term to test the U-shape of the moderated positive influence hypothesis and the moderated negative influence hypothesis. In addition, the wave-shaped pattern of our hypothesis that combines positive and negative influence was tested by including a cubic term for initial opinion discrepancy.

**Table 1:** Fixed Effects Estimates (Top) and Variance-Covariance Estimates (Bottom) for Models of the Predictors of Opinion Shifts after the First Stimulus in Study 1.

Parameter	Model 1	Model 2	Model 3
	Fixed effects		
Intercept	-4.35** (1.32)	-4.40** (1.48)	-5.71* (2.53)
Level 1 (observations)			
Distance	0.34 ***(0.09)	0.35* (0.18)	0.34* (0.17)
Distance^2^/100	-0.09 (0.14)	-0.12 (0.57)	-0.08 (0.56)
Distance^3^/10000		0.02 (0.47)	-0.01 (0.47)
Importance of issue			-0.79 (0.78)
Level 2 (participants)			
Gender (female = 1)			1.72 (1.47)
Year of study			0.75 (0.84)
Works (yes = 1)			1.48 (1.50)
	Random parameters		
Intercept var. *μ*_*0*_	21.49***	21.50***	20.87***
level-1 *σ*^*2*^	159.94	160.21	160.21
Model deviance	4943.56	4942.02	4928.76

*Notes*. N = 617 observations from 89 participants. Table shows restricted maximum likelihood HLM2 model estimates obtained in HLM 6. Numbers in parentheses are *robust* standard errors. For the variance of the random intercept, the *p*-value is obtained from a χ^2^-test.

* p<.05

** p<.01

*** p<.001

In Model 1 in Table 1, the *intercept* is negative and significant, showing that participants who perfectly agreed with the source shifted their opinions away from the source by 4.35 scale points on average. This shift away from the source, however, does not support the negative influence hypothesis, as the hypothesis predicts such shifts when individuals disagree with the source.

What is more important, the estimate of the distance parameter is positive and significant but the estimate of the squared distance is small and insignificant. This shows that, on average, opinion distance increased shifts towards the source linearly, which supports the linear positive influence hypothesis. The average opinion shifts that Model 1 of Table 1 implies are visualized in the right panel of Fig 1.

To test the positive and negative influence hypothesis, we included a cubic distance term in Model 2 of Table 1. This term, however, did not have a significant effect and including it failed to increase model fit, as the difference in model deviance between Models 1 and 2 was not significant (χ^2^(1) = 1.53, *p* = 0.22). Model 3 in Table 1 incorporates salience of the selected issue for the participant, gender [80], year of study, and labor market experience as controls. These variables did not have significant effects and failed to decrease model deviance significantly (χ^2^(4) = 13.26, *p* = 0.9899).

In sum, Study 1 supported the linear positive influence hypothesis. The more participants disagreed with the source, the more they were positively influenced, on average. We did not find limitations of positive influence when participants disagreed strongly with the source. Thus, we found no support for the moderated positive influence, the moderated negative influence, and the positive and negative influence hypotheses.

#### Effects on attraction

**Fig 2:**
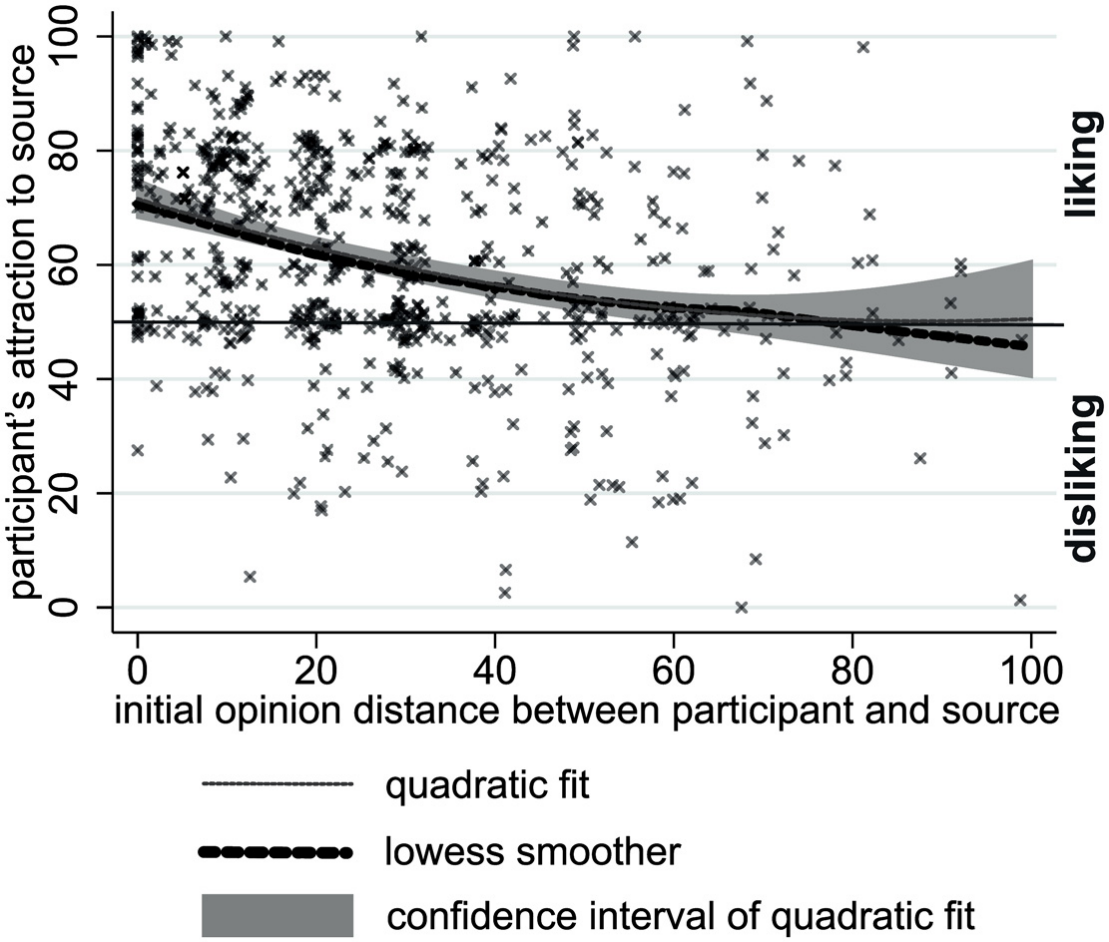
Relationship between initial opinion distance and attraction measured after the first stimulus in Study 1.

The average attraction towards the source was 61.3 (*SD* = 19.1) on an attraction scale that ranged from 0 to 100, suggesting that ratings were rather positive on average. 14.3% of the ratings, however, were below the center of the attraction scale, suggesting negative evaluations of the source. Fig 2 shows a descriptive analysis of the association between initial opinion discrepancy and attraction of the source. The average association of the two variables is indicated by the declining quadratic function that was fitted to the data. For large initial opinion distances, the fitted line does not significantly fall below the midpoint of the attraction scale, suggesting that even big opinion distances failed to create feelings of disliking on average.

The random intercept (multilevel) regression models of Table 2 provide the statistical test of the *attraction* and the *repulsion hypotheses*. In Model 1, we included only the intercept and a linear effect of opinion distance. The intercept is significantly higher than 50, which is the midpoint of the attraction scale. Opinion distance had a significantly negative effect on attraction. Its parameter estimate implies that attraction falls below the midpoint of the scale when opinion distance exceeds 68.3 scale points. However, as Model 2 shows and Fig 2 indicated, the effect of opinion distance on attraction is quadratic. The combination of the significant negative linear and positive quadratic effect of initial distance indicates that attraction rates decrease by opinion distance, but for large distances there is a slight increase. Most importantly, the estimated curve stays above the neutral attraction score of 50. This pattern supports the *attraction hypothesis*, but is inconsistent with the *repulsion hypothesis*. Including control variables did not affect this conclusion, but revealed that female participants gave higher attraction scores on average.

**Table 2:** Fixed Effects Estimates (Top) and Variance-Covariance Estimates (Bottom) for Models of the Predictors of Attraction Ratings after the First Stimulus in Study 1.

Parameter	Model 1	Model 2
	Fixed effects	
Intercept	69.81*** (1.67)	75.11*** (3.71)
Level 1(observations)		
Distance	-0.29*** (0.04)	-0.53*** (0.11)
Distance^2^/100		0.33** (0.12)
Salience		-1.43 (0.94)
Level 2 (participants)		
Gender (female = 1)		7.30** (2.58)
Works (yes = 1)		-3.72 (2.61)
Year of study		-0.69 (1.19)
	Random parameters	
Intercept var. *μ*_*0*_	109.73***	103.29***
level-1 *σ*^*2*^	223.98	219.74
Model deviance	5215.96	5189.04

*Notes*. N = 617 cases for 89 participants. Table shows restricted maximum likelihood HLM2 model estimates obtained in HLM 6. Numbers in parentheses are *robust* standard errors. The dependent variable “attraction” was measured on a 0…100 scale. For the variance of the random intercept, the *p*-value is obtained from a χ^2^-test. The improvement of model deviance between Model 2 and 1 is significant (χ^2^(5) = 27, *p* = 0.9999).

* p<.05

** p<.01

*** p<.001

### Discussion

Study 1 supported the linear positive influence hypothesis and the attraction hypothesis. It *did not support* the hypotheses of negative influence or the hypothesis of moderated positive influence. We found a small differentiation effect indicating a baseline tendency to move one’s opinion away from very similar opinions of others, but this tendency was combined with a linear and positive effect of opinion-distance on opinion shifts towards the source.

Even though neither of the negative influence hypotheses was supported, the results of Study 1 do not necessarily imply that there is no negative influence when subjects *dislike* the source. Therefore, we experimentally manipulated in Study 2 participants’ attraction towards the source.

## Study 2

### Procedure

The experiment took place in Groningen and lasted one hour per session. Participants were invited in groups of 10 and were randomly seated in cubicles. They were asked about their opinion and subjective importance attached to 20 issues at the beginning of the experiment (S1 Table).

Study 2 was different in two ways from Study 1. First, participants interacted repeatedly in real time with another participant so that both changes of opinion and of attraction could be measured. New software was developed to allow real-time computer-mediated communication between the participants in a pair. Participants were ensured that they interacted with partners present in the laboratory, which was indeed the case. The identity of partners was never revealed. The experiment was planned in such a way that every participant interacted nine times with one of the other participants.

Second, we manipulated interpersonal attraction in order to induce feelings of disliking towards the source of influence. In the *disliking treatment*, we used a combination of three existing methods to induce feelings of disliking towards the source: an assessment of subject of academic study, a regular Prisoner’s Dilemma task [47] and a choice between sending a stigmatizing or an overwhelmingly positive message to the interaction partner [47, 49]. In the disliking treatment, information about the partner’s choices or subject of study was displayed on the screen if the partner had a different subject of study, defected in the Prisoner’s Dilemma, or sent a stigmatizing message. Details of the manipulation can be found in S3 Text. In the control treatment, we did not manipulate attraction towards the current source of influence. Like in Study 1, participants were only informed about the opinion of their current interaction partner.

The two treatments were implemented in a within-subject design. All participants alternated between the two treatments, starting with the disliking treatment. Each interaction began with an initial measurement of attraction towards the source. After the initial opinion of the source was presented (*first stimulus*), opinion and attraction towards the source were measured again. In addition, in both treatments, persuasive messages were exchanged (*second stimulus*), and attraction and opinion were recorded again. Further details can be found in S4 Text.

Participants were matched with each other based on an algorithm that excluded issues with insufficient variation in initial opinions, and was designed to simultaneously increase the variance of initial opinion distances across pairs, the variance of distances to the opinion of the partner within the individual across matches, and to decrease the inequality of salience within pairs. The iterated algorithm selected 9 issues that provided solutions for these criteria and determined a random sequence among these issues.

### Participants

Participants were students from all faculties of the University of Groningen recruited with board advertisements, lecture announcements, and advertisements in the university newspaper. They were paid 8 Euros for their participation independently from their choices in the experiment. In addition, everybody had an equal chance to win 200 Euros in a lottery. Data from one session (N = 10) was excluded from the analysis due to missing values of the dependent variable, leading to a total of N = 100 participants. 90 participants interacted 9 times with another person of their session. Unfortunately, one experimental session was terminated already after 4 interactions due to technical problems. In total, we included 850 interactions in our analyses.

### Results

#### Manipulation check

The attraction manipulation was successful in decreasing attraction. After the first stimulus, the average attraction rating in the disliking treatment was above the midscale value (*M* = 56.57, *SD* = 21.85), but significantly lower than in the control treatment (*M* = 60.22, *SD* = 20.87; *t = 2*.*47*). In the control treatment, 16.3 percent of the attraction ratings were below the midpoint of the scale. In the disliking treatment, 26.0 percent of the ratings were below this point. For the disliking treatment, a multilevel linear regression of initial attraction (*M* = 53.83, *SD* = 21.64) yielded estimated effects (robust standard errors in brackets) of 60.57 (1.80)*** (intercept)– 7.01 (1.89)*** (*PairDefected*) –6.02 (1.73)*** (*Stigmatized*)– 1.72 (1.39) (*SameFaculty*). Hence, only the difference in study direction failed to decrease initial attraction. The manipulations based on behavior in the Prisoner’s Dilemma and on the stigmatizing task successfully decreased average attraction towards the partner, although it is not clear whether feelings of dislike were generated.

#### Effects on opinion shifts

Table 3 shows the relative frequency of positive and negative opinion shifts in the two treatments. Despite the decreased attraction ratings in the disliking treatment, the table shows no discernible difference in the occurrence of negative opinion shifts between treatments (*d = -0*.*56*, *t = -0*.*67* for the first shift, *d = -0*.*006*, *t = -0*.*007* after all stimuli).

**Table 3:** Relative Frequency of Positive, Negative, and No Opinion Shifts Observed in Study 2 after the First Stimulus and after All Stimuli.

	Control treatment	Disliking treatment
	After the first stimulus	
Positive shifts	34.74%	32.98%
No change	57.89%	58.30%
Negative shifts	7.37%	8.72%
	After all stimuli	
Positive shifts	54.21%	53.83%
No change	38.16%	36.81%
Negative shifts	7.63%	9.36%
N (100%)	380	470

In Table 4, we report random intercept (multilevel) multilevel regression models of opinion change after the first stimulus separately for the two treatments. The difference to the models estimated for Study 1 was that we added for the disliking treatment (Models 3–6) terms that allowed assessing the unique contribution of the independently manipulated initial attraction, as well as its potential interaction with initial opinion discrepancy.

**Table 4:** Fixed Effects Estimates (Top) and Variance-Covariance Estimates (Bottom) for Models of the Predictors of the First Opinion Shift in Study 2.

Parameter	Control treatment			Disliking treatment		
	Model 1	Model 2	Model 3	Model 4	Model 5	Model 6
	**Fixed effects**					
Intercept	-3.14***(0.88)	-1.19 (1.77)	-2.63** (0.83)	-3.20 (1.64)	1.34 (1.70)	-0.66 (2.16)
*Level 1 (observations)*						
Distance	0.64***(0.19)	0.63***(0.19)	0.44**(0.14)	0.44**(0.14)	0.29*(0.13)	0.29*(0.13)
Distance^2^/100	-1.53*(0.75)	-1.51*(0.74)	-0.72 (0.49)	-0.72 (0.49)	-0.67 (0.46)	-0.70 (0.46)
Distance^3^/10000	1.28 (0.71)	1.26 (0.70)	0.54 (0.42)	0.53 (0.41)	0.49 (0.38)	0.51 (0.38)
Initial attraction				0.01 (0.03)	-0.07* (0.03)	-0.07*(0.03)
Attraction * distance					0.003* (0.001)	0.003* (0.001)
Salience of issue		-0.20 (0.59)				0.77 (0.62)
*Level 2 (participants)*						
Gender (female = 1)		-0.82 (1.41)				1.16 (1.37)
Works (yes = 1)		-0.86 (1.36)				0.52 (1.10)
MA student (yes = 1)		-2.60*(1.28)				-0.31 (1.07)
	**Random parameters**					
Intercept var. *μ*_*0*_	15.65***	15.31***	0.77	0.85	0.85	0.46
level-1 *σ*^*2*^	100.95	101.17	129.13	129.28	127.64	128.43
Model deviance	2882.25	2869.33	3626.23	3629.80	3636.64	3626.66

*Notes*. N = 380 cases in the control treatment; N = 470 cases in the disliking treatment for 100 participants in each treatment. Missing values (N = 9) for the Works variable have been imputed by the overall mean (0.411).

* p<.05

** p<.01

*** p<.001

In the control treatment, as in Study 1, the opinion of the source was the only stimulus for shifting an opinion. Nevertheless, while in Study 1 we only found a strong positive linear effect of initial opinion discrepancy on the magnitude of positive opinion shift, Models 1 and 2 also have a significant *quadratic effect* of opinion distance in the control treatment in Study 2. This lends support for non-linear effects of initial discrepancy. Both Models 1 and 2 in Table 4 comprise significant effects of initial discrepancy (positive) as well as initial discrepancy squared (negative). Moreover, the cubic term of initial discrepancy in Model 2 has a positive effect that is close to being statistically significant at the *p* = 0.05 level.

**Fig 3:**
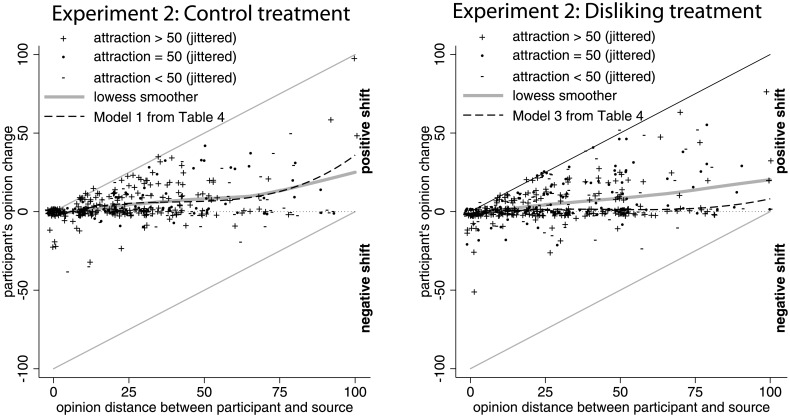
Visualization of observed opinion shifts in the control treatment and the disliking treatment of Study 2. In the right panel, the dashed line shows model prediction for the average initial attraction.

To facilitate interpretation, the left panel of Fig 3 plots Model 1 from Table 4, showing that the estimated curve remotely resembles the wave-shape predicted by the negative and positive influence hypothesis. Contrary to this hypothesis we find, however, that the estimated curve falls predominantly into the region of positive opinion shifts. This holds in particular for large initial opinion discrepancy, in contradiction with the negative and positive influence hypothesis. Despite the differences with Study 1, we conclude that also the results for the control treatment of Study 2 are well approximated by the linear positive influence hypothesis.

Likewise, also the disliking treatment supported the linear positive influence hypothesis, as Models 3, 4, 5, and 6 in Table 4 show. While initial opinion discrepancy had a significant and positive effect on opinion change, the non-linear effects of initial discrepancy were not significant. Moreover, also here the estimated curves fall predominantly into the region of positive opinion shifts (see right panel of Fig 3). As a further test, we estimated similar models also for the second opinion shift in the disliking treatment. Opinion shifts after the second stimulus were far smaller than shifts after the first stimulus. This is in line with the findings of decreasing message acceptance in persuasion research [63], but questions the assumption of constant weights in opinion dynamics models with repeated interactions [9: 282]. The smaller shifts after the second stimulus were in the same direction as first shifts and the impact of opinion distance has remained linear. These results clearly demonstrate that a longer interaction is not different qualitatively from a short influence process and it is the first impression that matters the most.

**Fig 4:**
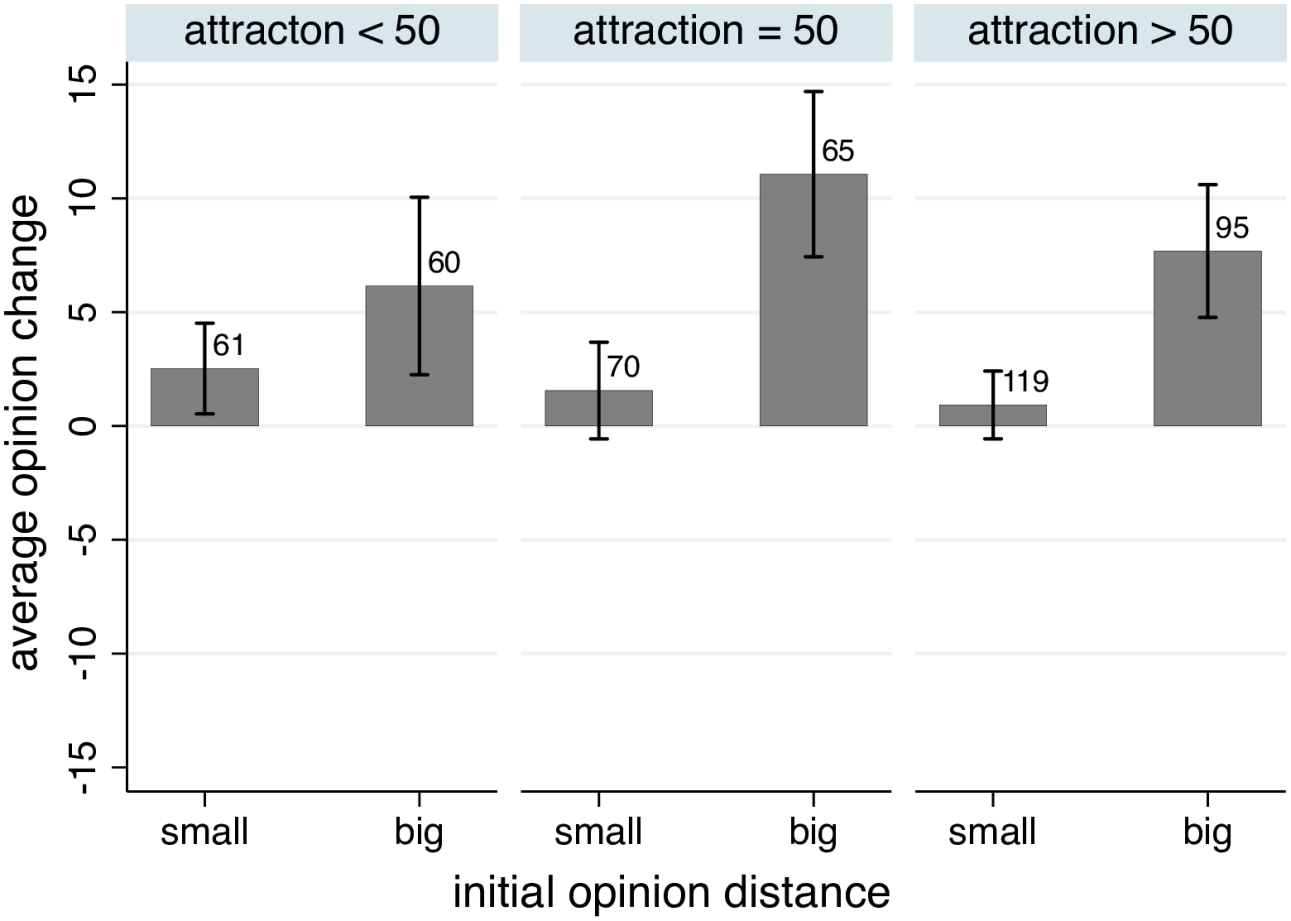
Average opinion shift after the first stimulus in the disliking treatment of Study 2, broken down by liking vs. disliking and small (below the mean of all) vs. large (above the mean of all) initial distances. The number of cases is displayed on top of the bars. Error bars show 95% confidence intervals.

The independent manipulation of attraction in the disliking treatment allowed us to test whether a low attraction induced negative influence independent of the effect of opinion distance. Models 5 and 6 in Table 4 have a significant *negative effect of attraction* on opinion change, which seems to contradict the hypothesis that disliking rather than liking reduces and even inverts positive opinion shifts. The contrast with Model 4 that does not include an interaction effect shows that the main effect of attraction needs to be interpreted in combination with the positive interaction effect of initial discrepancy with initial attraction. This implies that opinion shifts in case of distant and liked sources are attenuated, but when we take this into account, liking the source implies a net differentiation effect. As an additional illustration, Fig 4 compares the average opinion change by low, neutral, and high attraction towards the source, broken down by small vs. large initial opinion discrepancy. Overall, average opinion changes were positive in all categories. This pattern of results shows no indication of negative opinion shifts induced by disliking.

#### Effects on attraction

We tested the *attraction hypothesis* and the *repulsion hypothesis* with multilevel regression models of participants’ attraction towards the source (measured after the first stimulus). We estimated models separately for the two attraction treatments. In addition, we controlled in the regressions for initial attraction ratings. Table 5 displays the results.

**Table 5:** Fixed Effects Estimates (Top) and Variance-Covariance Estimates (Bottom) for Models of the Predictors of Attraction Ratings after the First Stimulus in Study 2.

Parameter	Control treatment		Disliking treatment	
	Model 1	Model 2	Model 3	Model 4
	Fixed effects			
Intercept	67.88***(1.84)	70.58***(4.71)	30.49***(3.14)	35.37***(5.46)
*Level 1(observations)*				
Distance	-0.28***(0.04)	-0.40***(0.11)	-0.20***(0.04)	-0.39**(0.13)
Distance^2^/100		0.17 (0.14)		0.10 (0.09)
Initial attraction			0.59***(0.04)	0.53***(0.08)
Attraction*distance				0.002 (0.002)
Salience of issue		-0.79 (1.20)		0.96 (0.93)
*Level 2 (participants)*				
Gender (female = 1)		1.77 (3.60)		1.13 (2.15)
Works (yes = 1)		-3.46 (3.26)		-2.61 (1.66)
MA student (yes = 1)		-1.36 (3.06)		-5.16**(1.56)
	Random parameters			
Intercept var. *μ*_*0*_	148.41***	152.01***	11.80	4.51
level-1 *σ*^*2*^	232.12	231.56	274.11	273.66
Model deviance	3270.89	3252.44	3993.86	3979.39

*Notes*. N = 360 cases in the control treatment and N = 450 cases in the disliking treatment for 100 participants in each treatment. The dependent variable is the attraction score measured after the first stimulus, which was the first attraction measurement in the control treatment and the second measurement in the disliking treatment.

* p<.05

** p<.01

*** p<.001

All models in Table 5 show that attraction ratings declined in initial opinion discrepancy. The large and positive intercept terms show that on average, participants liked the source of influence when initial discrepancy was small. Unlike in Study 1, the estimated attraction rating of the control treatment adopted values below the neutral point of 50 when the initial opinion distance exceeded a critical level. For maximum initial differences, predicted ratings dropped well below the mid-point of the scale of attraction, especially after controlling for background variables in Model 2. These results are consistent with the attraction hypothesis but also support the repulsion hypothesis.

In the disliking treatment, attraction was manipulated independently first. Initial attraction ratings have therefore been included in the analysis (Models 3 and 4 in Table 5). It turned out that attraction ratings determined the next measurement to a large extent. The significant positive intercept in Models 3 and 4 indicates a general tendency to evaluate the partner more positively after the first stimulus than before. Still, for large initial distances, estimated attraction ratings were below the midscale value, supporting the repulsion hypothesis.

## Summary and Conclusion

The negative influence hypothesis posits that opinion discrepancy or disliking of a source of an opinion elicits opinions shifts away from the position of the source. This hypothesis offers an explanation why stable bi-polarization of opinions can be observed in contexts in which positive social influence should lead to opinion convergence. Despite its prominent place in the literature, the negative influence hypothesis has hitherto not been submitted to experimental tests that provided conclusive evidence. We conducted two experimental studies in a highly controlled computer-mediated setting, in which we varied the initial opinion distance to and the liking or disliking of the source. With this approach we could avoid several weaknesses of previous experimental tests. We also add to a small but growing body of literature that aims to assess quantitatively the relationship between opinion differences, opinion change and characteristics of both source and target of influence in highly controlled experiments [81–83].

The results of our experiments challenge the negative influence hypothesis. Even when low attraction towards the source of influence was induced in Study 2, it did not trigger opinion shifts *away* from the opinion of the source. The strongest and most general effect we found was a positive linear effect of opinion distance on opinion shifts. A similar result for a social influence experiment in which subjects had to guess the right answer to a factual question has been reported by [81]. This finding implies for models of opinion dynamics that a complex non-linear social influence function might be unnecessary to characterize the relationship between similarity and opinion change. Our results suggest that not only for the sake of simplicity, but also for the sake of realism, model builders should be cautioned against resorting too readily to a more complex assumption than a simple linear influence function. This implies that exposure might provide the largest push towards consensus in case of large initial differences.

In fact, if negative opinion shifts occurred at all in our experiments, they did for small rather than large distances, potentially due to random shifts or because of a need for differentiation that some authors posited to be a driving force of social and cultural diversity [19, 84–90]. We found no support for the negative influence hypothesis in our data. Thus, to the extent that we can generalize this pattern towards a description of the micro-level process that drives social influence in larger scale repeated interactions between multiple individuals, our results do not yield an explanation of opinion polarization. It should be noted however, that models that assume tendencies for differentiation mainly for small distances can generate persistent opinion diversity—but not opinion polarization—in a larger population of interacting individuals under certain conditions [62].

While our experiments did not provide support of negative influence, we found evidence of another element of a possible explanation of opinion diversity, the attraction hypothesis. At the same time, there was only mixed evidence for repulsion in our data. In Study 1 we did not find support for the repulsion hypothesis, but in Study 2 we did. Large discrepancy induced less liking in Study 2, but contrary to theories of negative influence, this was not associated with negative opinion shifts. In short, our findings are only partly consistent with a motivation to reduce cognitive dissonance [39, 64–65] and with predictions of social judgment theory [40]. Individuals might have formed their opinions in a way to build and maintain a consistent system of beliefs and opinions, but this was not strictly differentiated based on liking of the source. Participants in our experiments tended to shift their opinions towards those of the source, irrespective of their evaluation. This might be because people in general do not easily distance away their opinion from others, even if the other one has an extreme position or is not very much liked. Yet a possible interpretation is that the most distant opinions were the most disturbing for the participants, but they reduced dissonance only by shifting opinions *towards* the position of the source, and never by *moving away* from it or by derogating the source. This might mean that despite the successful manipulations, “liking” and “disliking” in our experiments were not of crucial importance for opinion formation and cognitive balance.

There are four potential limitations concerning our studies and the generalizability of our results that point to avenues for future research. First, our participants were mainly students who could assume targets also to be students from the same university. This implicit similarity itself could explain the lack of discrepancy effect, particularly in Study 1. Second, the laboratory setting with computer-mediated anonymous interaction may have suppressed the emotional processes that induce disliking and negative influence in field settings. In case of face-to-face encounters, visible characteristics, sexual attraction, and facial expressions would be important variables that are difficult to measure and to control for. Our design excluded such factors on purpose, but the cost may have been that we could not observe some of the negative influence dynamics that might occur in field settings. We should not claim ex post, however, that our manipulations were “too weak”. These manipulations were not weak for positive social influence as half of the participants shifted their opinions towards the source as a result of receiving information about the opinion of their partner. The purpose of our experiment was not to induce negative influence, but to test hypotheses in simple and standard experimental situations to which important theories building on negative influence can also be applied.

Third, our experiments focused on dyadic social influence, as earlier experiments that exposed participants to multiple sources made it difficult to attribute opinion shifts to negative influence. This way we minimized group identification processes on purpose. According to theories based on social categorization, however, it may be those group identification processes that trigger negative influence. We deliberately excluded group-identification because many of the formal theories predicting bi-polarization based on a mix of positive and negative influence likewise exclude group memberships. Integrating group identities in an experimental framework testing negative influence is an important direction for future work.

Fourth, in roughly half of the cases, participants did not change their opinions at all and were biased towards round numbers on the attraction scale. One potential explanation for the lack of opinion change is “bounded confidence” [61, 91–93]. This theory suggests that large opinion discrepancies may result in a lack of any opinion change, rather than in negative shifts. Our data, however, does not seem to support this possibility, as the tendency to not change opinions was most likely for the smallest distances (e.g., *p* = 75.2% for the first quartile of the initial opinion distribution and *p* = 50.7% for the rest in Study 2, *z* = 6.66) and was not significantly different between middle and large opinion distances (e.g., *p* = 50.5% for the second quartile of the initial opinion distribution and *p* = 50.8% for third and fourth quartiles in Study 2, *z* = -0.06).

Furthermore, our analyses indicate that much of the variation in attraction ratings and opinion changes is not explained by initial discrepancy or attraction. Individuals seem to vary on how open they are to positive and negative influence and how intolerant they are for large inconsistencies of opinions [50, 94–97]. While we tried to capture some of this variation with individual level controls, future research could improve upon this and include measures of corresponding personality variables. Future research should also explore social influence on other kinds of opinions. We focused on issues without a true opinion value but with a moderately high salience to the participants. Nevertheless, it cannot be excluded that negative influence plays a role in communication about issues, where there is a true or socially desirable answer.

Overall, our experiments provided a highly controlled test of assumptions about negative social influence that have a prominent place in recent models of opinion formation. We find only very little evidence that these assumptions adequately describe the behavior of participants in the controlled settings of our experiments. We cannot exclude that negative influence may occur under other conditions in the laboratory or field. It would be interesting to identify the moderating factors that might lead to conclusions different from those we draw from our artificial settings. Our findings point to the need to inspect more carefully by which mechanisms and under what conditions negative influence is a plausible assumption.

## Supporting Information

S1 Datasets Datasets, codebooks, and STATA script for the analysis. (ZIP)

S1 Fig Observed shifts during Study 1 on the absolute opinion scale. The horizontal axis shows initial opinions on the 0…100 scale. The vertical axis charts opinion shifts. Positive values indicate a shift towards 100 and negative values towards 0. The grey solid lines define the bounds of possible opinion shifts.
(TIF)

S2 Fig Observed shifts during Study 2 on the absolute opinion scale. The horizontal axis shows initial opinions on the 0…100 scale. The vertical axis charts opinion shifts. Positive values indicate a shift towards 100 and negative values towards 0. The grey solid lines define the bounds of possible opinion shifts.
(TIF)

S1 Table Means and standard deviations of original opinions (O) and saliences (S) for issues used in the experiments. (DOCX)

S1 Text Further details of the procedure in Study 1. (DOCX)

S2 Text Details on the measurement of dependent variables. (DOCX)

S3 Text Details of the disliking manipulation in Study 2. (DOCX)

S4 Text Further details of the procedure in Study 2. (DOCX)
