# Supplementary material for: Discrepancy and Disliking Do Not Induce Negative Opinion Shifts
Source: PLoS One. 2016 Jun 22;11(6):e0157948. doi: 10.1371/journal.pone.0157948 (PMC4917087; doi:10.1371/journal.pone.0157948)
Supplement: S1 Datasets — (ZIP) [file pone.0157948.s001.zip › S1Datasets/Study1/CodebookStudy1corrected.pdf]

ICS
RUG/UU/KUN

INTERUNIVERSITY CENTER OF SOCIAL SCIENCE THEORY AND METHODOLOGY

Discrepancy and Disliking Do Not Induce Negative Opinion Shifts

Study 1

Principal Investigator:

Andreas Flache

Participating researchers:

Károly Takács

Michael Mäs

Department of Sociology

University of Groningen

Grote Rozenstraat 31

9712 TG Groningen

The Netherlands

Data set: Corevariables2labeled.sav

Background questionnaire: questionnaireStudy1.pdf

Debriefing of participants: debriefingEnglishStudy1.pdf, debriefingNetherlandsStudy1.pdf

Sponsor:

The research was supported by the Netherlands Organization for Scientific Research, NWO (AF, VIDI Grant 452-04-351, http://www.nwo.nl).

Publication based on the data:

Takács, K., Flache, A., & Mäs, M. (2016). Discrepancy and Disliking Do Not Induce Negative Opinion Shifts. *PLOS One*, under review.

Restriction of use:

All publications using these data, should cite the above paper and before its publication the following working paper:

Takács, K.; Flache, A., and Mäs, M. 2014. Is there negative social influence? Disentangling effects of dissimilarity and disliking on opinion shifts. arXiv:1406.0900 [physics.soc-ph] http://arxiv.org/abs/1406.0900

Corresponding author:
Károly Takács takacs.karoly@tk.mta.hu

Data collection method:
Study 1 is a laboratory experiment using a web-based questionnaire.

Units of analysis:
Observations (opinions) are nested in persons (participants), therefore the dataset has a multilevel character.

Weighting variables: -

Information on anonymizing: respondent id (rid) for respondents.

Procedure

The experiment took place at the University of Groningen and lasted 45 minutes per session. Participants were randomly seated in cubicles and responded to a web-based questionnaire. They first rated their opinions on and subjectively perceived importance of 31 issues. Next, for every participant an issue was selected. Participants were exposed to the opinion of the source on the selected issue (*first stimulus*). This opinion of the source was drawn from a pilot or an earlier session, which allowed using a web-based questionnaire without real online interactions. Further details about the procedure can be found in Text S1 of Takács, Flache, and Mäs (2016). As all pre-selected issues had a relatively high variance of opinions (Table S1 of Takács, Flache, and Mäs, 2016), our matching method ensured that we obtained a wide range of opinion similarity.

After the *first stimulus*, participants rated how much they liked the source (the other person) and gave their own opinion a second time on the same screen. Given the lack of any other information, participants could only base the attraction rating on the opinion of the source (the first stimulus). Participants' attraction towards the source and the difference between the second opinion measure and the opinion at the very beginning of the experiment constituted the two dependent variables of our analyses. Details of the measurement of the dependent variables can be found in Text S2 of Takács, Flache, and Mäs (2016).

Subsequently, participants received an updated opinion of the source on the issue (*second stimulus*, Text S1 of Takács, Flache, and Mäs, 2016), followed by another measurement of attraction and opinion change. Here, we focus on the effects of the first stimulus. The effects of the second stimulus were very much in line with those of the first, although the effects of the second stimulus turned out to be weaker.

For each participant, this procedure was repeated 7 to 9 times. Each time, a new source and a new issue was chosen. The order of issues followed an automated selection procedure that avoided repetition and spread issues evenly across different opinion positions. This manipulation created variance in opinion

distance between the target and source, which is the core independent variable of our study. At the end of the experiment, participants completed a questionnaire asking background data and motivations during the experiment.

Participants

Participants were 108 first and second year students of sociology at the University of Groningen (in the Netherlands) who participated as study requirement. After excluding all students from the pilot study part, *N*=89 participants were included in the analyses. As every participant was exposed to multiple sources, the total number of observed opinion exchanges was 617.

Response and non-response: varies between 0 and 90 (for opinion changes)

0 missing values	Rid<br>issueseq<br>salience<br>stimuli1<br>stimuli2<br>message<br>gender<br>year<br>work<br>conditio<br>distance<br>caseid<br>stchange<br>messages<br>mesoutof<br>mesappreci<br>meshappy<br>st1dis50<br>distsquare<br>distpower4<br>distpower3<br>opinion
60 missing values	attract<br>opinionc<br>opchange<br>newdist<br>distchge<br>overshot<br>sentmesa<br>sentmesb<br>sentmesc<br>dist2square<br>dist2pow4<br>dist2attr<br>dist2pow3<br>opsign<br>distsign
90missing values	netopch2<br>totalopi<br>attracti<br>totopch<br>opchang2<br>distanc2<br>dist2cha<br>topopsign
75 missing values	nochange

Description of variables and description of constructed/derived variables:
rid: Respondent id
issueseq: Issue sequence
salience: Salience of the issue: “How important is this question for you?”,
where 1: very important 2: important 3: unimportant 4: very unimportant.
stimuli1: First stimulus, the opinion of the source on the selected issue.
attract: The first measurement of attraction (as a result of the first stimulus), measured with the question “We would like to know your feelings about how much would you probably like this person?”,
where 0 means very much disliking and 100 means very much liking.
opinionc: opinion change on the 0…100 scale,
where negative number means that opinion has been shifted towards 0 and positive number means that opinion has been shifted towards 100.
opchange: Opinion shift after the first stimulus,
which is calculated as o_{i1}-o_{i2} if $o_{j1}\geq o_{i1}$ and as o_{i2}-o_{i1} otherwise for the first shift, and as o_{i1}-o_{iF} if $o_{j1}\geq o_{i1}$ and as o_{iF}-o_{i1} otherwise for the total shift; where o_{i1} is the original opinion of the subject, o_{j1} is the initial opinion of the source, o_{i2} is the opinion of the subject after the first stimulus, and o_{iF} is the final opinion of the subject.
stimuli2: Second stimulus, the updated opinion of the source on the selected issue.
message: received message,
where 1 means "“My opinion on this question is number y. You are out of grounds with your opinion. Your opinion is definitely not realistic. Rethink your position and take an opinion that is closer to y.” ,
2 means "“My opinion on this question is number y. I appreciate if you take an opinion that is closer to y.”,
3 means “My opinion on this question is number y. I am happy if you take an opinion that is closer to y.”,
4 means “My opinion on this question is number y. I might move closer to your opinion, if you take an opinion that is closer to y.”
netopch2: rough opinion change after 2nd stimulus.
totalopi: total opinion change on the 0…100 scale,
where negative number means that opinion has been shifted towards 0 and positive number means that opinion has been shifted towards 100 compared to the first stimulus.
attracti: attraction change
totopch: total opinion change after all stimuli
gender: gender of the respondent,
where 1 means female, 0 means male
year: year of studies,
where 1 means first year, 2 means second year, 3means third year and 4 means higher.
work: whether or not respondent has paid work at the moment,
where 1 means ‘Yes’, 0 means ‘No’ .

conditio: experimental session (A, B, C or D).
Note: A, B, C, D sessions were held on different days. All cases by a given subject, therefore, belong to one session: this variable should be handled as a group-level variable in any analysis. Opinion stimuli was independent of the session variable and varied between cases, hence this variable does not indicate the presence or absence of any experimental manipulation; it is just a nominal-scale variable for the date on which the experimental session took place.
distance: difference of initial opinion from first stimuli (to the opinion of the source on the selected issue).
caseid: case identifier
opchang2: Opinion shift after the second stimulus,
which is calculated as o_{i1}-o_{i2} if $o_{j1} \geq o_{i1}$ and as o_{i2}-o_{i1} otherwise for the first shift, and as o_{i1}-o_{iF} if $o_{j1} \geq o_{i1}$ and as o_{iF}-o_{i1} otherwise for the total shift; where o_{i1} is the original opinion of the subject, o_{j1} is the initial opinion of the source, o_{i2} is the opinion of the subject after the second stimulus, and o_{iF} is the final opinion of the subject.
distanc2: Absolute distance between 2nd stimulus and opinion before.
stchange: whether or not stimuli changed closer to original opinion,
where a positive value means change closer to original opinion,
a negative value means change further from original opinion,
0 means no change.
messages: created from the *message* variable,
where *mesoutof* means "My opinion on this question is number y. You are out of grounds with your opinion. Your opinion is definitely not realistic. Rethink your position and take an opinion that is closer to y.",
mesappreci means "My opinion on this question is number y. I appreciate if you take an opinion that is closer to y.",
meshappy means "My opinion on this question is number y. I am happy if you take an opinion that is closer to y."
and *mescloser* means "My opinion on this question is number y. I might move closer to your opinion, if you take an opinion that is closer to y." .
mesoutof: created from the *message* variable,
Received message: "My opinion on this question is number y. You are out of grounds with your opinion. Your opinion is definitely not realistic. Rethink your position and take an opinion that is closer to y.",
where 1 means 'Yes', 0 means 'No'.
mesappreci: created from *message* variable,
"My opinion on this question is number y. I appreciate if you take an opinion that is closer to y.",
where 1 means 'Yes', 0 means 'No'.
meshappy: created from *message* variable,
"My opinion on this question is number y. I am happy if you take an opinion that is closer to y.",
where 1 means 'Yes', 0 means 'No'.
newdist: distance between new opinion and first stimuli.

distchge: change of distance to stimuli1.
Opinion shift is calculated as x_1-x_2 (1st stimulus) and x_1-x_F (all stimuli), which is the difference between the original absolute distance to the source $x_1=|o_{i1}-o_{j1}|$ and the new absolute distance to the source's initial opinion $x_2=|o_{i2}-o_{j1}|$ and $x_F=|o_{iF}-o_{j1}|$, where o_{i1} is the original opinion of the subject, o_{j1} is the initial opinion of the source, o_{i2} is the opinion of the subject after the first stimulus, and o_{iF} is the final opinion of the subject.
overshot: overshooting, 0 if not, + if distance decreased, - if distance increased after first stimuli.
st1dis50: distance of stimulus1 from midpoint of scale 50.
dist2cha: change of absolute distance to second stimulus.
nochange: whether or not subject has changed his/her opinion,
where 1 means 'Yes', 0 means 'No'.
distsquare: distance square, initial / 100 .
distpower4: distance on the power of 4, initial / 1000000.
sentmesa: Sent message: "My opinion on this question is number y. You are out of grounds with your opinion. Your opinion is definitely not realistic. Rethink your position and take an opinion that is closer to y.",
where 1 means 'Yes', 0 means 'No'.
sentmesb: Sent message: "My opinion on this question is number y. I appreciate if you take an opinion that is closer to y.",
where 1 means 'Yes', 0 means 'No'.
sentmesc: Sent message: "My opinion on this question is number y. I am happy if you take an opinion that is closer to y."
where 1 means 'Yes', 0 means 'No'.
dist2square: created from *distance2* (absolute distance between 2nd stimulus and opinion before), as $distance2^2$ / 100.
dist2pow4: created from *distance2* (absolute distance between 2nd stimulus and opinion before), as $distance2^4$ / 1000000.
dist2attr: the interaction variable of *distance2* (absolute distance between 2nd stimulus and opinion before) and *attraction*.
distpower3: created from *initial* distance on the power of 3, divided by 10000.
dist2pow3: created from *distance2* (absolute distance between 2nd stimulus and opinion before), as distance2 on the power of 3, divided by 10000.
opchsign: created from *opchange*,
where +1 means positive shift as a result of the first stimulus, 0 means no change, and -1 means negative shift.
topchsign: created from *totopch*,
where +1 means positive shift as a result of all stimuli, 0 means no change, and -1 means negative shift.
distchsign: created from *distchge*,
where +1 means positive shift as a result of the first stimulus, 0: no change, -1: negative shift.
opinion: original opinion on the 0…100 scale.
