# Supplementary material for: Discrepancy and Disliking Do Not Induce Negative Opinion Shifts
Source: PLoS One. 2016 Jun 22;11(6):e0157948. doi: 10.1371/journal.pone.0157948 (PMC4917087; doi:10.1371/journal.pone.0157948)
Supplement: S1 Datasets — (ZIP) [file pone.0157948.s001.zip › S1Datasets/Study1/questionnaireStudy1.pdf]

Test-urennummer:
Respondent ID:
rid

ACHTERGROND-VRAGEN
BACKGROUND QUESTIONS

Rijks*universiteit* Groningen
Faculteit der Gedrags- en Maatschappijwetenschappen
Sociologie

Tot slot een aantal achtergrondvragen. Vul het juiste antwoord in, of kruis aan het antwoord dat van toepassing is.
By conclusion, here are a number of background questions. Please fill in the right answer or mark the answer that is appropriate with a cross.

1. Geslacht / Gender *gender*

    [ ] Man / Male

    [ ] Vrouw / Female

2. Geboortejaar / Year of birth: 19……………

Not included in the core dataset

3. Wat is je nationaliteit? / What is your nationality? ....

Not included in the core dataset

4. Wat is je moedertaal? / What is your native language?

    [ ] Nederlands / Dutch

    [ ] Anders,
    Namelijk / Other, namely: ……………………………….....

Not included in the core dataset

5. Waar ben je geboren? / Where have you born?

    [ ] In Nederland,
    in de provincie / In the Netherlands, in the province of

    ....

    [ ] Niet in Nederland,
    maar in / Not in the Netherlands, but in
    ………………………………………....
    Not included in the core dataset

6. Ben je religieus? / Are you religious?

    [ ] Nee. / No.

    [ ] Ja, namelijk / Yes,

    namely: [ ] Katholiek / Catholic

    [ ] Protestant / Protestant

    [ ] Moslim / Muslim

    [ ] Joods / Jewish

    [ ] Anders / Other

Not included in the core dataset

7. Hoeveelste jaars student ben je? / Which is the year of your study at the university?

[ ] Eerstejaars student / First-year student

[ ] Tweedejaars student / Second-year student

[ ] Derdejaars student / Third-year student

[ ] Hoger / Higher

year

8. Ben je lid van een studentenvereniging ? / Are you a member of a student organization?

[ ] Nee. (Ga door naar vraag 10.) / No. (Go to question 10.)

[ ] Ja. (Voortzet met vraag 9.) / Yes. (Continue with question 9.)

Not included in the core dataset

9. Indien je lid bent van een studentenvereniging:
Is het lid zijn van die studentenvereniging een belangrijk onderdeel van je identiteit?
/ In case you are a member of a student organization:
Being a member of this student organization is an important part of your identity?

Ja	Nee

Yes. No.
Not included in the core dataset

10. Heb je op het ogenblik betaald werk? / Do you have paid work at the moment?

Ja	Nee

Yes. No.
work

HARTELIJK DANK VOOR JE MEDEWERKING!!!!
THANK YOU VERY MUCH FOR YOUR HELP!!!
