# Supplementary material for: Discrepancy and Disliking Do Not Induce Negative Opinion Shifts
Source: PLoS One. 2016 Jun 22;11(6):e0157948. doi: 10.1371/journal.pone.0157948 (PMC4917087; doi:10.1371/journal.pone.0157948)
Supplement: S1 Datasets — (ZIP) [file pone.0157948.s001.zip › S1Datasets/Study2/CodebookStudy2.pdf]

ICS
RUG/UU/KUN
INTERUNIVERSITY CENTER OF SOCIAL SCIENCE THEORY AND METHODOLOGY

Discrepancy and Disliking Do Not Induce Negative Opinion Shifts

Study 2

Principal Investigator:
Andreas Flache
Participating researchers:
Károly Takács
Michael Mäs

Department of Sociology
University of Groningen
Grote Rozenstraat 31
9712 TG Groningen
The Netherlands

Data set: Allshift2labeled.sav

The software for Study 2 was developed by Vincent Hindriksen in Delphi.

Script used for Study 2: mergedscriptforStudy2.rtf

Sponsor:
The research was supported by the Netherlands Organization for Scientific Research, NWO (AF, VIDI Grant 452-04-351, http://www.nwo.nl).

Publication based on the data:

Takács, K., Flache, A., & Mäs, M. (2016). Discrepancy and Disliking Do Not Induce Negative Opinion Shifts. *PLOS One*, under review.

Restriction of use:
All publications using these data, should cite the above paper and before its publication the following working paper:

Takács, K.; Flache, A., and Mäs, M. 2014. Is there negative social influence? Disentangling effects of dissimilarity and disliking on opinion shifts. arXiv:1406.0900 [physics.soc-ph] http://arxiv.org/abs/1406.0900

Corresponding author:
Károly Takács takacs.karoly@tk.mta.hu

Data collection method:
Study 2 is a laboratory experiment.

Units of analysis:
Observations (opinions) are nested in persons (participants), therefore the dataset has a multilevel character.

Weighting variables: -

Information on anonymizing: respondent id (id) for respondents.

Procedure

The experiment took place in Groningen and lasted one hour per session. Participants were invited in groups of 10 and were randomly seated in cubicles. They were asked about their opinion and subjective importance attached to 20 issues at the beginning of the experiment (Table S1 of Takács, Flache, and Mäs, 2016).

Study 2 was different in two ways from Study 1. First, participants interacted repeatedly in real time with another participant so that both changes of opinion and of attraction could be measured. New software was developed to allow real-time computer-mediated communication between the participants in a pair. Participants were ensured that they interacted with partners present in the laboratory, which was indeed the case. The identity of partners was never revealed. The experiment was planned in such a way that every participant interacted nine times with one of the other participants.

Second, we manipulated interpersonal attraction in order to induce feelings of disliking towards the source of influence. In the *disliking treatment*, we used a combination of three existing methods to induce feelings of disliking towards the source: an assessment of subject of academic study, a regular Prisoner's Dilemma task (Sampson and Insko, 1964) and a choice between sending a stigmatizing or an overwhelmingly positive message to the interaction partner (Sampson and Insko, 1964; Schwartz and Ames, 1977). In the disliking treatment, information about the partner's choices or subject of study was displayed on the screen if the partner had a different subject of study, defected in the Prisoner's Dilemma, or sent a stigmatizing message. Details of the manipulation can be found in Text S3 of Takács, Flache, and Mäs (2016). In the control treatment, we did not manipulate attraction towards the current source of influence. Like in Study 1, participants were only informed about the opinion of their current interaction partner.

The two treatments were implemented in a within-subject design. All participants alternated between the two treatments, starting with the disliking treatment. Each interaction began with an initial measurement of attraction towards the source. After the initial opinion of the source was presented (*first stimulus*), opinion and attraction towards the source were measured again. In addition, in both treatments,

persuasive messages were exchanged (*second stimulus*), and attraction and opinion were recorded again. Further details can be found in Text S4 of Takács, Flache, and Mäs (2016).

Participants were matched with each other based on an algorithm that excluded issues with insufficient variation in initial opinions, and was designed to simultaneously increase the variance of initial opinion distances across pairs, the variance of distances to the opinion of the partner within the individual across matches, and to decrease the inequality of salience within pairs. The iterated algorithm selected 9 issues that provided solutions for these criteria and determined a random sequence among these issues.

Participants

Participants were students from all faculties of the University of Groningen recruited with board advertisements, lecture announcements, and advertisements in the university newspaper. They were paid 8 Euros for their participation independently from their choices in the experiment. In addition, everybody had an equal chance to win 200 Euros in a lottery. Data from one session (N=10) was excluded from the analysis due to missing values of the dependent variable, leading to a total of N=100 participants. 90 participants interacted 9 times with another person of their session. Unfortunately, one experimental session was terminated already after 4 interactions due to technical problems. In total, we included 850 interactions in our analyses.

References

Sampson EE, Insko CA. Cognitive consistency and performance in the autokinetic situation. J Abnorm Soc Psychol, 1964; 68, 184-192. DOI: 10.1037/h0041242

Schwartz SH, Ames RE. Positive and Negative Referent Others as Sources of Influence: A Case of Helping. Sociometry, 1977; 40:12-21.

Takács, K., Flache, A., & Mäs, M. (2016). Discrepancy and Disliking Do Not Induce Negative Opinion Shifts. *PLOS One*, under review.

Response and non-response: varies between 0 and 278 (for opinion changes)

0 missing values	caseid<br>id<br>session<br>gender<br>totalnetshift<br>inidist2<br>inidist4<br>indistat<br>socscistudent<br>dist1expost4<br>shift1sign<br>shifttsign<br>inidist3<br>inidistance<br>dist1<br>firstshift<br>finaldist<br>totalshift<br>totalopchange<br>salience	MAstudent<br>dist1expost<br>dist2<br>secondshift<br>mesoutof<br>mesappreci<br>meshappy<br>recmeshappy<br>recmesoutof<br>recmesappreci<br>dist1expost2<br>distattraction<br>stchange<br>dist1expost3<br>condition<br>Period<br>issuenumber<br>iniopinion<br>pairid<br>iniopinionpair	attraction<br>opinionmod<br>message<br>recmessage<br>recopinion<br>attraction2<br>opinion2<br>message2<br>recmessage2<br>recopinion2<br>attraction3<br>opinion3<br>credible<br>faculty<br>pd<br>stigmasent<br>work
40 missing values	rndissue<br>attractelse<br>opinelse<br>underst<br>mood<br>know<br>intent<br>selfcont<br>exhaust<br>invloed<br>recipr<br>msginf<br>consist<br>bestans<br>svo1<br>svo2<br>svo3<br>svo4<br>sharesy<br>sharepair	risk1<br>risk2<br>risk3<br>risk4<br>stlevel<br>rel<br>lang<br>prov<br>edufath<br>edumoth<br>assoc<br>ident<br>expbefor	

278<br>missing<br>values	pairfaculty<br>pairpd<br>pairstigma<br>facultysame		

Description of variables and description of constructed/derives variables:
caseid: decision id
id: respondent id
session: session id
Subject: subject id within the session.
studies: studies at the university (RUG), where 1 means ‘Yes’, 0 means ‘No’.
rndissue: id of randomly selected issue for a vignette in the questionnaire.
attractelse: attraction toward vignette pair in questionnaire.
opinelse: updated opinion on vignette question after influence.
underst: How well did you understand the tasks during the experiment?, where 0 means ‘Completely’, 1 means ‘Well', 2 means 'Only the basics', 3 means 'Not really' and 4 means 'not at all’ .
mood: Which figure would describe your mood the best at the moment?', where 0 means ' :))', 1 means ':) ', 2 means ':|' 3 means ':(' and 4 means ':((‘.
know: Have you recognized any other participants in this experiment?, where 0 means 'I have not seen the other participants clearly.', 1 means 'I have seen the other participants, but have not recognized any.', 2 means 'I have recognized 1 or 2 other participants.' and 3 means 'I have recognized 3 or more other participants.'.
intent: Please indicate which answer indicates the best your intention during the experiment, where 0 means 'I was giving random answers', 1 means 'I was giving sincere answers purposefully', 2 means 'I was giving false answers purposefully'.
selfcont: Do you usually prefer to work things out yourself than get someone else’s advice?, where 0 means 'I always prefer to work things out myself', 1 means 'I rather prefer to work things out myself', 2 means 'I rather prefer to get someone else’s advice' and 3 means 'I always prefer to get someone else’s advice'.
exhaust: How exhausting was the experiment for you?, where 0 means 'very exhausting', 1 means 'exhausting', 2 means 'bit exhausting only', 3 means ‘not exhausting' and 4 means 'refreshing'.
invloed: Please indicate how important were these factors, when you revealed your opinion during the experiment.: the opinion of the other participant, where 0 means 'very important', 1 means 'important', 2 means 'neutral', 3 means 'not important' and 4 means 'not important at all'.
recipr: Please indicate how important were these factors, when you revealed your opinion during the experiment.: the change in the opinion of the other participant, where 0 means 'very important', 1 means 'important', 2 means 'neutral', 3 means 'not important' and 4 means 'not important at all'.

msginf: Please indicate how important were these factors, when you revealed your opinion during the experiment.: the message of the other participant', where 0 means 'very important', 1 means 'important', 2 means 'neutral', 3 means 'not important' and 4 means 'not important at all'.
consist: Please indicate how important were these factors, when you revealed your opinion during the experiment.: to be consistent with my opinions in other issues, where 0 means 'very important', 1 means 'important', 2 means 'neutral', 3 means 'not important' and 4 means 'not important at all'.
bestans: Please indicate how important were these factors, when you revealed your opinion during the experiment.: what is expected by others to be the best answer', where 0 means 'very important', 1 means 'important', 2 means 'neutral', 3 means 'not important' and 4 means 'not important at all'.
svo1, svo2, svo3, svo4: 'In the next questions we ask you to imagine that you are paired with another person, who is not in this room and you do not know. We will refer to this person simply as the 'Other'. You will have to make choices that produce money rewards for you and for the 'Other.'.
svo1: 0 means that the first choice is 'I receive 24 euros and the other receives 2 euros.'
1 means that the first choice is 'I receive 29 euros and the other receives 14 euros.'
2 means that the first choice is 'I receive 26 euros and the other receives 21 euros.'
svo2: 0 means that the second choice is 'I receive 27 euros and the other receives 4 euros.'
1 means that the second choice is 'I receive 29 euros and the other receives 14 euros.'
2 means that the second choice is 'I receive 28 euros and the other receives 19 euros'.
svo3: 0 means that the third choice is 'I receive 25 euros and the other receives 25 euros.'
1 means that the third choice is 'I receive 26 euros and the other receives 20 euros.'
2 means that the third choice is 'I receive 25 euros and the other receives 15 euros.'
svo4: 0 means that the forth choice is 'I receive 34 euros and the other receives 19 euros.'
1 means that the forth choice is 'I receive 29 euros and the other receives 7 euros.'
2 means that the forth choice is 'I receive 31 euros and the other receives 27 euros'.
sharesy: Assume that you have won 100 euro. Other participants, who are in this room, did not have luck and they did not win anything. Please indicate how much money would you give *from your reward* to the following person:
sharepair: Assume that you still have your 100 euros entirely. Please indicate how much money would you give *from your reward* to the following person: with whom you have been paired.
risk1, risk2, risk3, risk4: In the next questions, we kindly ask you to choose between two options. Please select the option, which you *would* like to receive more, than the other. If you think that the first option is exactly as good for you, as the second, then please select the 'Equally good' button.
risk1: 0 means that the first chosen option is 'You will RECEIVE 26 euros for sure.'
1 means that the first chosen option is 'A fair coin will be thrown. You will RECEIVE 15 euros, if heads are thrown and you will RECEIVE 37 euros, if tails are thrown.'
2 means that the first chosen option is 'Equally good'.
risk2: 0 means that the second chosen option is 'You will RECEIVE 26 euros for sure', 1 means that the second chosen option is 'A fair coin will be thrown. You will RECEIVE nothing, if heads

are thrown and you will RECEIVE 52 euros, if tails are thrown', 2 means that the second chosen option is 'Equally good' .

risk3: 0 means that the third chosen option is 'You will LOSE 26 euros for sure.', 1 means that the third chosen option is 'A fair coin will be thrown. You will LOSE 15 euros, if heads are thrown and you will LOSE 37 euros, if tails are thrown.', 2 means that the third chosen option is

risk4: 0 means that the forth chosen option is 'You will LOSE 26 euros for sure.', 1 means that the forth chosen option is 'A fair coin will be thrown. You will LOSE nothing, if heads are thrown and you will LOSE 52 euros, if tails are thrown.', 2 means that the forth chosen option is 'Equally good'.

gender: gender of the respondent, where 1 means 'Female', 0 means 'Male' .

stlevel: At which level do you study?, where 0 means 'BA' 1 means 'Master' 2 means 'Ph.D' and 3 means 'other'.

rel: Are you religious? If yes, namely:, where 0 means 'No', 1 means 'Catholic', 2 means 'Protestant', 3 means 'Muslim', 4 means 'Jewish' and 5 means 'Other'.

lang: native language of the respondent, where 0 means 'Dutch', 1 means 'Frisian', 2 means 'Dutch and Frisian' and 3 means 'Other' .

prov: province of birth, where 0 means 'Groningen', 1 means 'Friesland', 2 means 'Drenthe', 3 means 'Flevoland', 4 means 'Overijssel', 5 means 'Gelderland', 6 means 'Noord-Holland', 7 means 'Zuid-Holland', 8 means 'Utrecht', 9 means 'Brabant', 10 means 'Limburg', 11 means 'Zeeland' and 12 means 'Not in the Netherlands' .

edufath: highest education of the respondents father, where 0 means 'Primary school, basic education, or a part of these', 1 means 'Lower vocational education; LTS, LHNO, Leao, retail (vocational) school', 2 means 'General secondary school; ULO, MULO, MAVO. Middleschool', 3 means 'Secondary vocational education; MTS, UTS, MBA, MEAO', 4 means 'general secondary school; HBS, Gymnasium, Lyceum, HAVO', 5 means 'Higher professional education; HTS, HEAO, HBO', 6 means 'Tertiary education', 7 means 'Other' and 8 means 'I don't know' .

edumoth: highest education of the respondents mother, where 0 means 'Primary school, basic education, or a part of these', 1 means 'Lower vocational education; LTS, LHNO, Leao, retail (vocational) school', 2 means 'General secondary school; ULO, MULO, MAVO. Middleschool', 3 means 'Secondary vocational education; MTS, UTS, MBA, MEAO', 4 means 'general secondary school; HBS, Gymnasium, Lyceum, HAVO', 5 means 'Higher professional education; HTS, HEAO, HBO', 6 means 'Tertiary education', 7 means 'Other' and 8 means 'I don't know' .

assoc: member of a student association, where 1 means 'Yes', 0 means 'No'.

ident: if member of a student organisation: is the membership of the student organisation an important part of your identity?, where 1 means 'Yes', 0 means 'No'.

work: whether or not respondent has paid work at the moment, where 1 means 'Yes', 0 means 'No'.

expbefor: has the respondent ever participated in a similar experiment before?, where 1 means 'Yes', 0 means 'No'.

condition: experimental condition, where 1 means 'opinion is presented first' and 2 means 'attraction is manipulated first'.

Period: interaction number in the experiment.
issuenumber: the selected issue number.
iniopinion: initial opinion on the issue.
pairid: id of the respondent's pair
iniopinionpair: initial opinion of the respondent's pair.
attraction: initial attraction after receiving the opinion of the pair on the selected issue, measured with the question "We would like to know your feelings about how much would you probably like this person?".
opinionmod: first updated opinion.
message: First sent message, where 0 means "My opinion on this question is number y. You are out of grounds with your opinion. Your opinion is definitely not realistic. Rethink your position and take an opinion that is closer to y.", 1 means "My opinion on this question is number y. I appreciate if you take an opinion that is closer to y.", 2 means "My opinion on this question is number y. I am happy if you take an opinion that is closer to y.", 3 means "My opinion on this question is number y. I might move closer to your opinion, if you take an opinion that is closer to y.".
recmessage: First received message, where 0 means "My opinion on this question is number y. You are out of grounds with your opinion. Your opinion is definitely not realistic. Rethink your position and take an opinion that is closer to y.", 1 means "My opinion on this question is number y. I appreciate if you take an opinion that is closer to y.", 2 means "My opinion on this question is number y. I am happy if you take an opinion that is closer to y.", 3 means "My opinion on this question is number y. I might move closer to your opinion, if you take an opinion that is closer to y.".
recopinion: received opinion.
attraction2: attraction towards the partner after the first stimulus.
opinion2: opinion on the issue after the first stimulus.
message2: Second sent message, where 0 means "My opinion on this question is number y. You are out of grounds with your opinion. Your opinion is definitely not realistic. Rethink your position and take an opinion that is closer to y.", 1 means "My opinion on this question is number y. I appreciate if you take an opinion that is closer to y.", 2 means "My opinion on this question is number y. I am happy if you take an opinion that is closer to y.", 3 means "My opinion on this question is number y. I might move closer to your opinion, if you take an opinion that is closer to y.".
recmessage2: Second received message, where 0 means "My opinion on this question is number y. You are out of grounds with your opinion. Your opinion is definitely not realistic. Rethink your position and take an opinion that is closer to y.", 1 means "My opinion on this question is number y. I appreciate if you take an opinion that is closer to y.", 2 means "My opinion on this question is number y. I am happy if you take an opinion that is closer to y.", 3 means "My opinion on this question is number y. I might move closer to your opinion, if you take an opinion that is closer to y.".
recopinion2: second received opinion.

attraction3: attraction towards the partner after the second stimulus.
opinion3: opinion on the issue after the second stimulus.
credible: To what extent do you think the person you're paired with is a credible person?, where 0 means ‘Very credible’, 1 means ‘Credible’, 2 means ‘Neutral’ 3 means ‘Incredible’ 4 means ‘Very incredible’.
faculty: respondent’s faculty, where 0 means 'Management', 1 means 'Economics', 2 means 'Behavioral and social sciences', 3 means 'Theology and religious studies', 4 means 'arts', 5 means 'medical sciences', 6 means 'spatial sciences', 7 means 'law', 8 means 'philosophy', 9 means 'natural sciences' , 99 means 'does not study at the university'.
pd: respondent's decision, where 0 means 'I give away the bonus', 1 means 'I keep the bonus' .
stigmasent: whether or not stigmatizing message sent, where 1 means ‘Yes’, 0 means ‘No’.
pairfaculty: Pair’s faculty, where 0 means 'Management', 1 means 'Economics', 2 means 'Behavioral and social sciences', 3 means 'Theology and religious studies', 4 means 'arts', 5 means 'medical sciences', 6 means 'spatial sciences', 7 means 'law', 8 means 'philosophy', 9 means 'natural sciences' , 99 means 'does not study at the university'.
pairpd: pair’s decision, where 0 means 'I give away the bonus', 1 means 'I keep the bonus' .
pairstigma: whether or not stigmatizing message sent from partner.
iniattraction: attraction after the attraction manipulation.
inidistance: initial distance between initial opinion and the partner’s initial opinion,measured as abs(iniopinion – iniopinionpair).
dist1: distance after first stimulus, measured as abs (opinionmod – iniopinionpair).
firstshift: first absolute change of distance to iniopinionpair, measured as inidistance – dist1.
finaldist: final absolute distance to last observed opinion of other, measured as abs(opinion3 – recopinion2).
totalshift: total change of distance to last observed opininon of other, measured as inidistance – finaldist.
totalopchange: total opinion change (net, initial - final opinion), measured as iniopinion – opinion3.
salience: Salience of issue: How important..?, where 1 means 'very important', 2 means 'important', 3 means 'unimportant', 4 means 'very unimportant' .
MAstudent: created from stlevel, 1 if stlevel=1, otherwise 0
Master student (highest study years), where 1 means ‘Yes’, 0 means ‘No’.
dist1expost: distance at first stimulus, measured as abs(opinionmod - recopinion)
dist2: the absolute distance between opinion after the first stimulus and received opinion, measured as abs (opinion2 - recopinion).
secondshift: opinion shift due to 2second stimulus, the difference between distance at first stimulus and the absolute distance between opinion after the first stimulus and received opinion, measured as dist1expost – dist2.
mesoutof: created from message, if message = 0 then mesoutof = 1, Sent message: “My opinion on this question is number y. You are out of grounds with your opinion. Your opinion is

definitely not realistic. Rethink your position and take an opinion that is closer to y.", where 1 means 'Yes', 0 means 'No'.
mesappreci: created from message, if message = 1 then mesapprec = 1, Sent message: "My opinion on this question is number y. I appreciate if you take an opinion that is closer to y.", where 1 means 'Yes', 0 means 'No'.
meshappy: created from message, if message = 2 then meshappy = 1, Sent message: "My opinion on this question is number y. I am happy if you take an opinion that is closer to y.", where 1 means 'Yes', 0 means 'No'.
recmeshappy: created from recmessage, if recmessage = 2 then meshappy = 1
Received message: "My opinion on this question is number y. I am happy if you take an opinion that is closer to y.", where 1 means 'Yes', 0 means 'No'.
recmesoutof: created from recmessage, if recmessage = 0 then mesoutof = 1
Received message: "My opinion on this question is number y. You are out of grounds with your opinion. Your opinion is definitely not realistic. Rethink your position and take an opinion that is closer to y.", where 1 means 'Yes', 0 means 'No'.
recmesappreci: created from recmessage, if recmessage = 1 then mesapprec = 1, Received message: "My opinion on this question is number y. I appreciate if you take an opinion that is closer to y.", where 1 means 'Yes', 0 means 'No'.
dist1expost2: created from dist1expost as dist1expost * dist1expost / 100.
distattraction: interaction between dist1expost and attraction, measured as dist1expost * attraction.
stchange: change of stimulus, positive if closer to initial opinion, measured as abs(ininopinionpair – iniopinion) – abs(recopinion – iniopinion).
facultysame: respondent and pair attend the same faculty, where 1 means 'Yes', 0 means 'No'.
totalnetshift: total shift compared to initial opinion of pair,measured as inidistance – abs(opinion3 – iniopinionpair).
inidist2: created from inidistance as inidistance * inidistance / 100.
inidist4: created from inidistance as $inidistance^4$ / 1000000.
indistat: interaction between inidistance and iniattraction,measured as inidistance * iniattraction (relevant for Condition II).
socscistudent: social science (gmw/ppsw) student,where 1means 'Yes', 0 means 'No'.
dist1expost4: created from dist1expost as $dist1expost^4 / 1000000$.
shift1sign: sign of firstshift, where -1 means negative shift, 0 means no change and 1 means positive shift.
shifttsign: sign of totalnetshift, where -1 means negative shift, 0 means no change and 1 means positive shift.
inidist3: created from inidistance as $inidistance^3$ / 10000.
dist1expost3: created from dist1expost as $dist1expost^3$ / 10000
