# Supplementary figures and images for: Discrepancy and Disliking Do Not Induce Negative Opinion Shifts
Source: PLoS One. 2016 Jun 22;11(6):e0157948. doi: 10.1371/journal.pone.0157948 (PMC4917087; doi:10.1371/journal.pone.0157948)

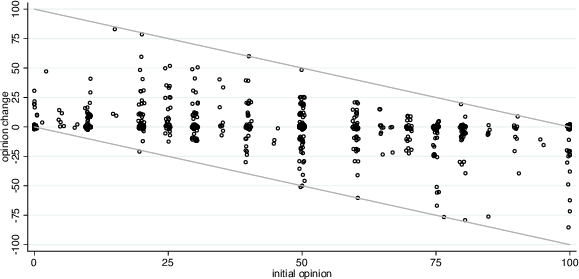

Supplement: S1 Fig — The horizontal axis shows initial opinions on the 0…100 scale. The vertical axis charts opinion shifts. Positive values indicate a shift towards 100 and negative values towards 0. The grey solid lines define the bounds of possible opinion shifts. (TIF) [file pone.0157948.s002.tif]

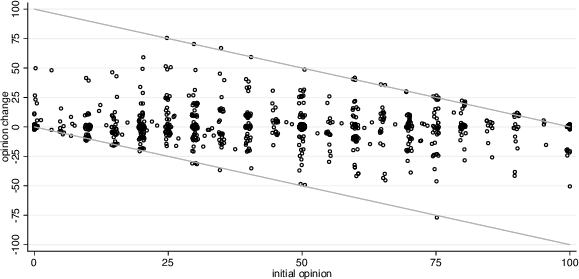

Supplement: S2 Fig — The horizontal axis shows initial opinions on the 0…100 scale. The vertical axis charts opinion shifts. Positive values indicate a shift towards 100 and negative values towards 0. The grey solid lines define the bounds of possible opinion shifts. (TIF) [file pone.0157948.s003.tif]
