# Supplementary material for: Discrepancy and Disliking Do Not Induce Negative Opinion Shifts
Source: PLoS One. 2016 Jun 22;11(6):e0157948. doi: 10.1371/journal.pone.0157948 (PMC4917087; doi:10.1371/journal.pone.0157948)
Supplement: S1 Table — (DOCX) [file pone.0157948.s004.docx]

**Table S1. Means and standard deviations of original opinions (O) and saliences (S) for issues used in the experiments**

| Issues | Study 1 (N=89) | | Study 2 (N=110) | |
| --- | --- | --- | --- | --- |
|  | O | S | O | S |
| 1. The warning signs on cigarette boxes should cover 0…100 percent of the box total surface. | 43.4 (29.5) | 2.04 (.78) | 44.5 (29.5) | 2.20 (.88) |
| 2. Smoking should be allowed at 0…100 percent of tables in café’s. | 32.8 (27.5) | 1.71 (.57) | 23.6 (27.1) | 1.75 (.75) |
| 3. The introduction of the euro brings advantages and disadvantages to us. 0…100 percent of all effects are advantages. | 51.4 (23.3) | 2.16 (.74) | 55.6 (24.9) | 2.30 (.76) |
| 4. The government should subsidize public transport in 0...100 percent. | 69.8 (22.5) | 1.56 (.58) | 65.7 (22.4) | 1.65 (.60) |
| 5. A demonstration needs police protection. Organizers should pay 0…100% of the costs of this. | 51.1 (33.1) | 2.21 (.67) | 44.0 (34.0) | 2.35 (.67) |
| 6. 0…100 percent of immigrants who come to the Netherlands for economic reason should receive a residence permit. | 34.8 (29.6) | 1.83 (.63) | 36.3 (29.6) | 1.82 (.68) |
| 7. Foreigners who want a residence permit for the Netherlands should pay 0…100 percent of their integration courses and tests. | 43.0 (33.5) | 1.92 (.79) | 42.2 (35.2) | 1.96 (.81) |
| 8. 0...100 percent of the streets in the centre of Groningen should have security camera surveillance. | 32.0 (27.1) | 2.15 (.70) | 33.7 (31.1) | 2.17 (.78) |
| 9. Sport activities of students should be financed by the university in 0…100% of total costs. | 49.9 (26.3) | 2.18 (.67) | 52.2 (27.8) | 2.20 (.81) |
| 10. Universities should be financed in 0…100 percent by tuition fees. | 40.0 (22.5) | 1.91 (.67) | 36.7 (22.0) | 2.03 (.64) |
| 11. The final grade of the overall study should be determined in 0…100 percent by the result of the Master’s thesis. | 41.9 (21.9) | 2.13 (.73) | 32.6 (20.3) | 2.35 (.71) |
| 12. Somebody who is caught to draw graffiti should pay a fine of 0...100 euro. | 49.9 (28.6) | 2.47 (.66) | 52.0 (28.1) | 2.52 (.62) |
| 13. The fine for not cleaning up after your dog making dirt on the street should be 0…100 euro. | 43.3 (31.5) | 2.28 (.77) | 38.6 (25.8) | 2.25 (.64) |
| 14. The CEO of an industrial company has a limited budget for building a new plant. This budget has to be divided between employing additional employees and investing in measures that protect the environment. 0…100 percent of the available budget should go to environment-protecting measures. | 48.4 (20.5) | 1.88 (.72) | 45.7 (20.7) | 1.83 (.59) |
| 15. The government has to divide an available budget between two options: building new highways or new high-speed railway tracks. 0…100 percent of these resources should be used to build new high-speed railway tracks. | 62.0 (20.4) | 1.88 (.64) | 65.8 (19.2) | 1.84 (.57) |
| 16. Schools in disadvantaged areas should receive 0…100 percent more financing than schools that are not in disadvantaged areas. | 33.0 (19.7) | 1.85 (.59) | 34.9 (21.2) | 1.80 (.54) |
| 17. The Dutch military has to divide 100 million euros between activities within the national borders, such as national defense and training, and missions outside the country. Foreign missions should receive 0…100 million euros. | 45.1 (20.2) | 2.22 (.65) | 45.4 (22.5) | 2.39 (.85) |
| 18. Students should pay 0...100 percent of the costs of language courses offered by the university. | 36.6 (25.0) | 2.29 (.79) | 33.7 (26.2) | 2.17 (.78) |
| 19. Foreign students should pay 0...100 percent of their Dutch language courses. | 42.8 (27.1) | 2.26 (.73) | 31.5 (28.3) | 2.17 (.73) |
| 20. Students should spend a maximum of 0…100 percent of the 40 hours weekly working time on paid work. | 41.2 (27.4) | 2.34 (.80) | 52.8 (32.4) | 2.54 (.81) |
| 21. In a family with two children, in which the husband works and the wife stays at home, the wife should take 0…100 percent of the household duties. | 71.9 (12.6) | 2.15 (.68) |  |  |
| 22. The local government faces two alternatives for extending the amount of housing: to build on non-residential areas or to rebuild, renovate or extend existing buildings. 0…100 percent of the resources should be devoted to build on non-residential areas. | 30.2 (21.0) | 2.03 (.73) |  |  |
| 23. The government should finance propagation of modern poetry in 0…100 percent of costs. | 20.4 (24.5) | 3.01 (.79) |  |  |
| 24. The government should pay 0...100 percent of the costs of child day-care. | 56.9 (25.0) | 1.73 (.56) |  |  |
| 25. A new shopping mall is built. The costs of building a street to the shopping mall has to be paid in 0…100 percent by the shopping mall. | 49.6 (26.8) | 2.51 (.62) |  |  |
| 26. A maximum of 0…100 percent of the total EU budget should be spent on agriculture. | 23.7 (15.1) | 2.27 (.75) |  |  |
| 27. Israel should pay 0…100 percent of the costs of rebuilding in Lebanon. | 56.5 (28.4) | 1.93 (.70) |  |  |
| 28. For the problems around integration in the Netherlands the responsibility goes in 0…100 percent to Muslims (0 = the responsibility is in 0% of Muslims, and in 100% is of non-Muslims). | 38.9 (26.7) | 1.84 (.88) |  |  |
| 29. A professor at the university should spend 0…100 percent of his or her working time on teaching (thus not on research and not on administrative duties). | 45.0 (21.2) | 2.27 (.73) |  |  |
| 30. The final grade of a subject should be determined in 0…100 percent by a result of a written exam. | 71.3 (17.9) | 2.02 (.71) |  |  |
| 31. Students should spend 0…100 percent of the 40 hours weekly working time on their study. | 64.3 (19.9) | 2.06 (.82) |  |  |

*Notes:* All issues were measured on a 0…100 percentage scale. Salience was measured on an ordinal scale: “How important…” with answer categories “very important”=1, “important”=2, “unimportant”=3, “very unimportant”=4. Formulations are independent back-translations from Dutch.
