# Supplementary material for: Discrepancy and Disliking Do Not Induce Negative Opinion Shifts
Source: PLoS One. 2016 Jun 22;11(6):e0157948. doi: 10.1371/journal.pone.0157948 (PMC4917087; doi:10.1371/journal.pone.0157948)
Supplement: S1 Text — (DOCX) [file pone.0157948.s005.docx]

**S1 Text. Further details of the procedure in Study 1**

Participants were reminded that all opinions they receive are from real people. After the sessions, participants have received an e-mail about the aim of the experiment and how exactly this has been achieved. This contained the following text: “…Consequently, participants were matched with other subjects, who in fact were from earlier sessions. This was due to technical reasons. (By using different software, we will be able to achieve in the following experiments that every participant is matched with another participant in the same session.)”

After the first stimulus, participants rated how much they liked the source (the other person) and gave their own opinion a second time on the same screen. Subsequently, participants were informed about the updated opinion of the source on the issue (*second stimulus*), followed by another measurement of attraction and opinion change. The updated opinion of the source was identical to the first stimulus (53.8%), was shifted by 5 units from the first stimulus (24.7%), and was shifted by 10 units from the first stimulus (21.5%). These shifts were taken from the pilot and were all small, as we did not intend to measure reactions to large opinion changes of the source.
