# Supplementary material for: Discrepancy and Disliking Do Not Induce Negative Opinion Shifts
Source: PLoS One. 2016 Jun 22;11(6):e0157948. doi: 10.1371/journal.pone.0157948 (PMC4917087; doi:10.1371/journal.pone.0157948)
Supplement: S2 Text — (DOCX) [file pone.0157948.s006.docx]

**S2 Text. Details on the measurement of dependent variables**

Opinion shifts were calculated as the difference between the original absolute distance to the source |*o_i1_-o_j1_*| and the new absolute distance to the initial opinion of the source |*o_i2_-o_j1_*| and |*o_iF_-o_j1_*|, where *o_i1_* was the original opinion of the participant, *o_j1_* was the initial opinion of the source, *o_i2_* was the opinion of the participant after the first stimulus, and *o_iF_* was the final opinion of the participant.
