# Supplementary material for: Discrepancy and Disliking Do Not Induce Negative Opinion Shifts
Source: PLoS One. 2016 Jun 22;11(6):e0157948. doi: 10.1371/journal.pone.0157948 (PMC4917087; doi:10.1371/journal.pone.0157948)
Supplement: S3 Text — (DOCX) [file pone.0157948.s007.docx]

**S3 Text. Details of the disliking manipulation in Study 2**

A difficulty of our design was that to induce feelings of disliking towards the source in an ethical and sincere way. In the experiments of [47], dislike was induced by a long procedure involving a failed cooperation task and an insult from the other party. In the experiments of [49], a negative referent was offending the subject. Keeping this in mind and in order to have sufficient variation in attraction ratings, we used a combination of three different methods in the *disliking treatment*.

First, at the beginning of the experiment, we assessed every participant’s subject of academic study. Participants had to pick from a menu at which faculty they study. The options were: management; economics; behavioral and social sciences; theology and religious studies; arts; medical sciences; spatial sciences; law; philosophy; natural sciences; does not study at the university. The justification for this manipulation was that students often develop their social identity based on their subject of study and distinguish themselves from others with a different study.

Second, participants had to select a strategy against their partner in a regular Prisoner’s Dilemma task. Instructions were:

Assume that you receive 10 euro as a bonus. You have to decide:

- whether you give away your bonus
- or you keep your bonus.

The other participant faces the same decision as you. If you give away your bonus and the other participant keeps it, then (s)he receives his or her own bonus and receives also yours. If you both decide to give away your bonuses, both of you receive 15 euro. This is summarized in the following table:

Table 1. Your rewards (euro):

| Your decision \| Decision of the other participant | (s)he gives the bonus away | (s)he keeps the bonus |
| --- | --- | --- |
| You give your bonus away | 15 | 0 |
| You keep your bonus | 20 | 10 |

Third, participants chose between sending a stigmatizing or an overwhelmingly positive message to their interaction partner. The two possible messages were formulated as: 1. “I am a very nice person. I will do all my best to help you and nobody else in this experiment.”; 2. “You have to know that I want to do my best in this experiment and I do not care about what you are going to receive.”

Participants learned that their decisions might be (but not necessarily will be) displayed on the screen of their partner. In the disliking treatment, information about the partner’s choices or subject of study was displayed on the screen if the partner had a different subject of study, defected in the Prisoner’s Dilemma, or sent a stigmatizing message. The screen remained empty when the partner studied the same subject, cooperated, and sent a friendly message.
