# Supplementary material for: Discrepancy and Disliking Do Not Induce Negative Opinion Shifts
Source: PLoS One. 2016 Jun 22;11(6):e0157948. doi: 10.1371/journal.pone.0157948 (PMC4917087; doi:10.1371/journal.pone.0157948)
Supplement: S4 Text — (DOCX) [file pone.0157948.s008.docx]

**S4 Text. Further details of the procedure in Study 2**

After the initial opinion of the source was presented (*first stimulus*), opinion and attraction towards the source were measured. Subsequently, in both treatments, participants sent one from a list of four persuasive messages to the other person. Subjects could select from the following messages: 1 = “My opinion on this question is number y. You are out of grounds with your opinion. Your opinion is definitely not realistic. Rethink your position and take an opinion that is closer to y.”; 2 = “My opinion on this question is number y. I appreciate if you take an opinion that is closer to y.”; 3 = “My opinion on this question is number y. I am happy if you take an opinion that is closer to y.”; 4 = “My opinion on this question is number y. I might move closer to your opinion, if you take an opinion that is closer to y.”

In the following screen, along with the opinion of the other person, the persuasive message received was displayed (*second stimulus*), and attraction and opinion were recorded again.
